# Identification of druggable host dependency factors shared by multiple SARS-CoV-2 variants of concern

**DOI:** 10.1093/jmcb/mjae004

**Published:** 2024-02-01

**Authors:** Ilaria Frasson, Linda Diamante, Manuela Zangrossi, Elena Carbognin, Anna Dalla Pietà, Alessandro Penna, Antonio Rosato, Ranieri Verin, Filippo Torrigiani, Cristiano Salata, Marìa Paula Dizanzo, Lorenzo Vaccaro, Davide Cacchiarelli, Sara N Richter, Marco Montagner, Graziano Martello

**Affiliations:** D epartment of Molecular Medicine, University of Padua, Padua 35121, Italy; D epartment of Molecular Medicine, University of Padua, Padua 35121, Italy; Department of Biology, Armenise/Harvard Pluripotent Stem Cell Biology Laboratory, University of Padua, Padua 35131, Italy; D epartment of Molecular Medicine, University of Padua, Padua 35121, Italy; Department of Biology, Armenise/Harvard Pluripotent Stem Cell Biology Laboratory, University of Padua, Padua 35131, Italy; Department of Surgery, Oncology and Gastroenterology, University of Padua, Padua 35128, Italy; Department of Surgery, Oncology and Gastroenterology, University of Padua, Padua 35128, Italy; Department of Surgery, Oncology and Gastroenterology, University of Padua, Padua 35128, Italy; Veneto Institute of Oncology IOV-IRCCS, Padua 35128, Italy; Department of Comparative Biomedicine and Food Science, University of Padua, Padua 35020, Italy; Department of Comparative Biomedicine and Food Science, University of Padua, Padua 35020, Italy; D epartment of Molecular Medicine, University of Padua, Padua 35121, Italy; D epartment of Molecular Medicine, University of Padua, Padua 35121, Italy; Telethon Institute of Genetics and Medicine (TIGEM), Armenise/Harvard Laboratory of Integrative Genomics, Pozzuoli 80078, Italy; Department of Translational Medicine, University of Naples Federico II, Naples 80138, Italy; Telethon Institute of Genetics and Medicine (TIGEM), Armenise/Harvard Laboratory of Integrative Genomics, Pozzuoli 80078, Italy; Department of Translational Medicine, University of Naples Federico II, Naples 80138, Italy; School for Advanced Studies, Genomics and Experimental Medicine Program, University of Naples Federico II, Naples 80138, Italy; D epartment of Molecular Medicine, University of Padua, Padua 35121, Italy; Microbiology and Virology Unit, Padua University Hospital, Padua 35128, Italy; D epartment of Molecular Medicine, University of Padua, Padua 35121, Italy; Department of Biology, Armenise/Harvard Pluripotent Stem Cell Biology Laboratory, University of Padua, Padua 35131, Italy

**Keywords:** SARS-CoV-2, variants of concern, host dependency factors, antivirals, N-acetyl cysteine

## Abstract

The high mutation rate of SARS-CoV-2 leads to the emergence of multiple variants, some of which are resistant to vaccines and drugs targeting viral elements. Targeting host dependency factors, e.g. cellular proteins required for viral replication, would help prevent the development of resistance. However, it remains unclear whether different SARS-CoV-2 variants induce conserved cellular responses and exploit the same core host factors. To this end, we compared three variants of concern and found that the host transcriptional response was conserved, differing only in kinetics and magnitude. Clustered regularly interspaced short palindromic repeats screening identified host genes required for each variant during infection. Most of the genes were shared by multiple variants. We validated our hits with small molecules and repurposed the US Food and Drug Administration-approved drugs. All the drugs were highly active against all the tested variants, including new variants that emerged during the study (Delta and Omicron). Mechanistically, we identified reactive oxygen species production as a key step in early viral replication. Antioxidants such as N-acetyl cysteine (NAC) were effective against all the variants in both human lung cells and a humanized mouse model. Our study supports the use of available antioxidant drugs, such as NAC, as a general and effective anti-COVID-19 approach.

## Introduction

The continuous emergence of multiple variants is an intrinsic feature of the SARS-CoV-2 pandemic. This poses significant hurdles to the development of prophylactic approaches, as new variants can partially overcome the immunity generated by COVID-19 vaccines (Krause et al., 2021). Currently, two antivirals are used to treat COVID-19, Paxlovid (containing nirmatrelvir and ritonavir) and Veklury (containing remdesivir), following approval by European Medicines Agency and US Food and Drug Administration (FDA). However, targeting viral proteins eventually leads to the selection of resistant variants (Kozlov, 2022). For example, it has recently been reported that treatment with nirmatrelvir, an inhibitor of 3CL^pro^, the major protease of SARS-CoV-2, leads to the emergence of resistant mutants (Hu et al., 2023; Iketani et al., 2023). Inhibition of host factors may be a better strategy to avoid resistance, as host factors are not under selective pressure to favour viral replication. Currently, besides the observed differences in transmissibility and clinical severity, we do not know whether different variants share the same life cycle or use different molecular pathways within the host cell: in the first case, new variant-independent druggable targets could be envisaged. Unfortunately, a direct comparison among different variants is still lacking due to the variety of model systems (cell lines, organoids, and transgenic animals) and variants used in different studies (Takayama, 2020).

Previous genetic screens on a different combination of virus variants and host cells highlighted the roles of several cellular proteins in SARS-CoV-2 infection but had little overlap (Daniloski et al., 2020; Baggen et al., 2021; Schneider et al., 2021; Wang et al., 2021b; Zhu et al., 2021). It is still unclear whether this was due to real heterogeneity in the cellular proteins exploited by different variants for their replication or due to technical differences. Here, we analysed the transcriptional responses of human lung cells to three SARS-CoV-2 variants that sequentially overtook each other during the pandemic, namely the Wuhan, D614G, and Alpha variants, and performed a high-stringency clustered regularly interspaced short palindromic repeats (CRISPR)-based genetic loss-of-function screen to identify host factors required for these variants. Among the identified 525 genes, most were shared by two or more variants. Gene Ontology (GO) analysis revealed enrichment of terms related to mitochondrial organization and oxidative stress. By performing short interfering RNA (siRNA)-mediated candidate gene knockdown and targeting selected proteins with currently available drugs for potential COVID-19 repurposing, we found that RIPK4, SLC7A11, and MASTL were implicated in virus-induced cytotoxicity, which was also validated on two additional variants (Delta and Omicron) that emerged during our study. Finally, we focused on SLC7A11, a cystine transporter belonging to the solute carrier family 7 that plays a crucial role in regulating the cellular redox state, and demonstrated that disrupting the accumulation of virus-induced reactive oxygen species (ROS) impeded viral replication. Taken together, our study identified the targets and compounds that may be effective against current and future variants of SARS-CoV-2.

## Results

### Transcriptome analysis of Calu-3 cells upon infection with three SARS-CoV-2 variants

To study the biology of SARS-CoV-2 and its interactions with human host cells, we selected three major SARS-CoV-2 variants of concern that emerged and spread globally by the end of 2020. The Wuhan variant is the original strain that emerged in late 2019. The D614G variant emerged shortly thereafter, in early 2020, and contains the indicated mutation that is retained in all the later variants and thought to enhance viral replication. The Alpha variant (B.1.1.7), first detected in December 2020, contains several mutations in the Spike protein, making it more infectious (up to 50% more transmissible) and associated with increased disease severity compared to the original Wuhan strain (Supplementary Table S1). As the respiratory tract is the initial and primary infection site for all SARS-CoV-2 variants, we used human lung epithelial Calu-3 cells, which are both susceptible (i.e. expressing both the ACE2 receptor and the TMPRSS2 cofactor on the cell membrane) and permissive (i.e. allowing efficient viral replication with the production of high levels of viral progeny, leading to cell death) to SARS-CoV-2 infection (Tseng et al., 2005; Hoffmann et al., 2020; Rosli et al., 2021). Other human lung cell lines are susceptible but only marginally permissive (Wang et al., 2021a). Alternative cell lines were rendered susceptible by expressing ACE2 via plasmid transfection (Hoffmann et al., 2020, 2021). In the latter two cases, no detectable cytopathic effect on virus release was observed.

Calu-3 cells were infected with the Wuhan, D614G, and Alpha strains at a multiplicity of infection (MOI) of 0.1, and the amounts of intracellular viral RNA and new infective viral particles were measured at 24 and 48 hours post infection (h.p.i.) (Supplementary Figure S1A and B). The Wuhan variant produced the fewest viral transcripts but most abundant new infective particles, the Alpha variant produced abundant viral transcripts but few new infective particles, and the D614G variant produced both viral transcripts and particles abundantly. In addition, both parameters increased strongly from 24 to 48 h.p.i. for Wuhan and D614G variants but remained constant for the Alpha variant. Thus, the three variants differed in viral transcription and replication rates (Hou et al., 2020; Zhou et al., 2021; de Souza et al., 2022; Tang et al., 2022; Supplementary Figure S1A and B). Notably, all three variants caused Calu-3 cell death within 48 h.p.i. (Kim et al., 2021; Rosli et al., 2021; Guarnieri et al., 2023), among which the Alpha variant acted more quickly, leading to complete cell death by 24 h.p.i., consistent with its faster viral transcript production.

**Figure 1:**
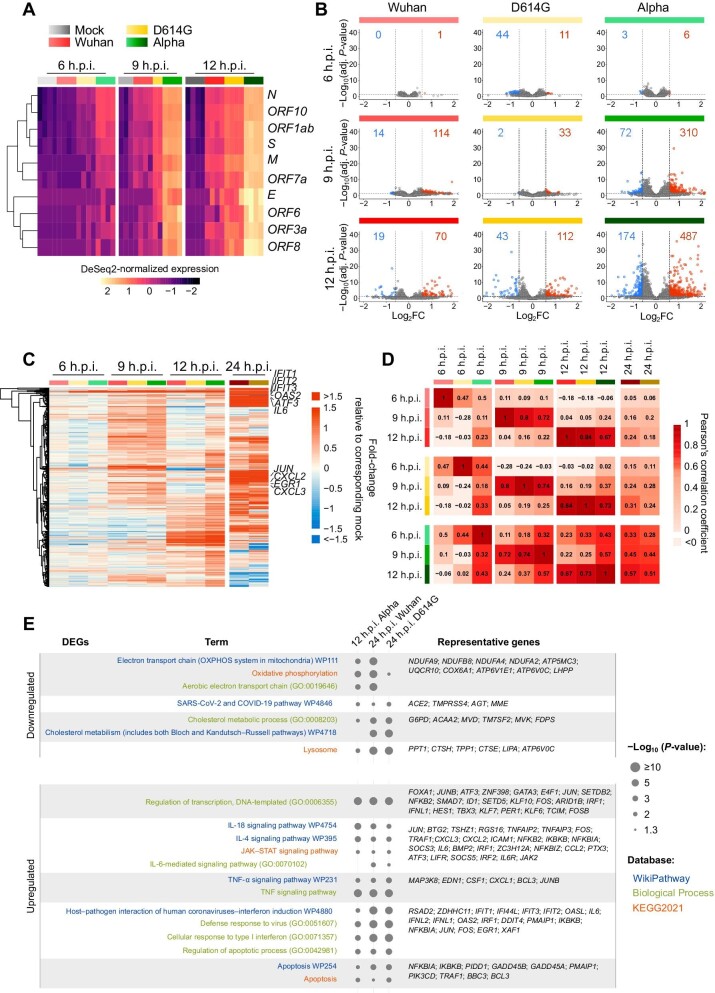
Different SARS-CoV-2 variants induce highly similar transcriptional responses. (**A**) Heatmap of viral transcripts in uninfected cells (mock) or cells infected with the indicated variants. The expression levels of viral transcripts are shown as row-scaled *Z*-scores. (**B**) Volcano plots showing DEGs (log_2_FC>0.59 or <−0.59, adj. *P*-value <0.05, vs. uninfected cells) in cells infected with the indicated variants at the indicated time points. The numbers of upregulated and downregulated DEGs are indicated in red and blue, respectively. FC, fold-change; adj. *P*-value, adjusted *P*-value. (**C**) Heatmap of DEGs in cells infected as indicated. (**D**) Correlation matrix displaying Pearson's correlation coefficients among the indicated samples. (**E**) Gene enrichment analysis of DEGs.

We next investigated both viral and cellular transcriptome changes at 6, 9, 12, and 24 h.p.i. (during the first cycle of viral replication) via RNA sequencing (RNAseq). To maximize the number of infected cells and thus avoid confounding data from uninfected cells, we increased the MOI to 1. Since the Alpha variant caused complete cell death at 24 h.p.i., no data could be collected from this condition. We found that viral transcripts were readily detectable upon infection with D614G and Alpha variants for 6 h and, in general, the Alpha variant had the most pronounced transcriptional response, followed by D614G and Wuhan variants (Figure 1A). These findings were confirmed by quantitative real-time polymerase chain reaction (qPCR; Supplementary Figure S1C and D) and consistent with those obtained at lower MOIs (Supplementary Figure S1A and B).

Simultaneously, we analysed the expression levels of cellular transcripts and found the number of differentially expressed genes (DEGs) starting increasing at 9 h.p.i. (Figure 1B). Similarly, the Alpha variant induced the greatest degree of variation in cellular gene expression, followed by D614G and Wuhan variants (Figure 1B). Most of the DEGs exhibited similar patterns across all the variants, except that the Alpha variant induced stronger and quicker responses (Figure 1B and C). Notably, gene expression patterns at 24 h.p.i. with Wuhan and D614G variants were similar to that at 12 h.p.i. with the Alpha variant (Figure 1C). Several of the modulated genes, including *IL6*, *IFIT1/2/3*, *OAS2*, *CXCL2*, *ATF3*, and *EGR1*, have previously been reported to be induced by SARS-CoV-2 infection both *in vitro* and in patients (Blanco-Melo et al., 2020; Wyler et al., 2021). Differences in *EGR1* and *ATF3* transcriptional response kinetics and magnitude upon infection with different SARS-CoV-2 variants were confirmed via qPCR (Supplementary Figure S1D).

To further investigate the similarities in cellular transcriptional responses, we calculated the correlation coefficients among different samples (Figure 1D). A value of 1 reflects identical gene expression levels of two compared samples. As shown in Figure 1D, the weak correlations among samples from 6 h.p.i. were consistent with the absence of a robust transcriptional response, while the increased correlation coefficients among samples at 9 and 12 h.p.i. with different variants indicated the convergence toward similar gene expression profiles. Importantly, the comparison with publicly available transcriptomic data revealed a highly significant overlap of DEGs across different coronavirus variants and species (Supplementary Figure S2A). To analyse cellular functions of the DEGs, we performed a gene enrichment analysis (Figure 1E). The downregulated genes were associated with a cellular environment characterized by reduced mitochondrial respiration, decreased cholesterol synthesis, and decreased expression of ACE2 and TMPRSS4, suggesting that these genes are crucial mediators of SARS-CoV-2 infection. Conversely, cellular processes such as gene transcription, interferon induction, interleukin signalling pathway, JAK–STAT signalling pathway, TNF signalling pathway, and the response to human coronaviruses were significantly activated, in line with previous findings (Blanco-Melo et al., 2020; Kim et al., 2021; Sanders et al., 2021; Wyler et al., 2021; Guarnieri et al., 2023). Finally, apoptosis-related genes were upregulated, consistent with the cell death caused by the Alpha variant infection.

### High-stringency CRISPR-based loss-of-function screen in Calu-3 cells infected with three SARS-CoV-2 variants

**Figure 2:**
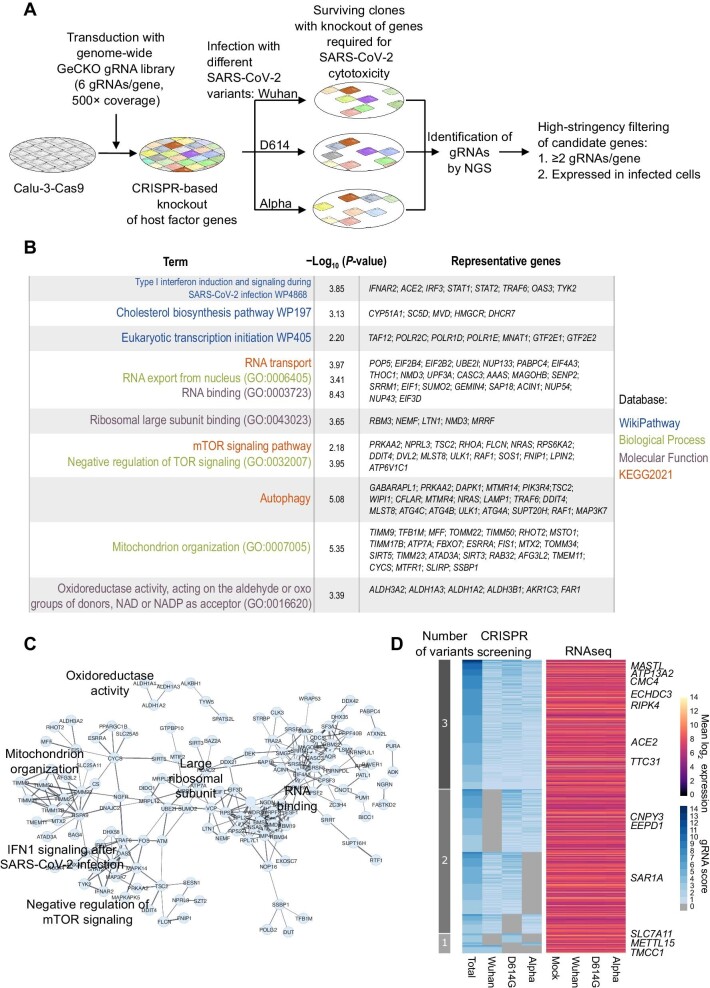
High-stringency CRISPR-based loss-of-function screen provides biological insights into different SARS-CoV-2 variants. (**A**) Schematics of the screening strategy. (**B**) Gene enrichment analysis of the hits identified by CRISPR screen. (**C**) Protein network of the screen hits, generated by Stringdb. Edge width is proportional to the strength of the interaction (see Supplementary Materials and methods). (**D**) Left: a heatmap displaying gRNA scores of candidate genes for each variant or across all three variants. Right: mean expression levels of candidate genes at 12 h.p.i., shown as log_2_-normalized expression.

The transcriptome analysis demonstrated a significant similarity in both viral and cellular transcription patterns among the three variants, with variations predominantly observed at the temporal level. However, this analysis did not provide insights into the host dependency factors that are shared among different variants. Previous studies identified host genes required for SARS-CoV-2 replication with very little overlap (Daniloski et al., 2020; Baggen et al., 2021; Schneider et al., 2021; Wang et al., 2021b; Zhu et al., 2021; Supplementary Figure S2B), probably due to the use of different cell lines and viral strains. Therefore, we attempted to make a systematic and direct comparison among the three variants by performing a high-stringency CRISPR-based loss-of-function screen in Calu-3 cells under identical experimental conditions (Figure 2A; Supplementary Figure S2C). To this end, Cas9-expressing Calu-3 cells were stably transduced with a genome-wide guide RNA (gRNA) library to direct knockout of a single gene per cell. The integrated gRNA served both as a guide for Cas9 and as a barcode for identifying the target locus. These Calu-3 cells were infected with SARS-CoV-2 at an MOI of 3, which resulted in complete cell death within 48 h.p.i., as determined in non-transduced cells. Genomic DNA was extracted from uninfected cells and surviving clones after infection for gRNA identification by next-generation sequencing (NGS), and the ‘gRNA score’ was determined for each SARS-CoV-2 variant or across all variants (see Materials and methods for details). To reduce false-positive and off-target effects, we ensured high coverage (500×, in triplicate for each variant) and excluded gRNAs targeting genes at low expression levels.

Initially, we focused on genes with the gRNA score >1 (Supplementary Table S2) but removed those not consistently expressed in Calu-3 cells. Gene enrichment analysis identified terms previously associated with SARS-CoV-2 infection, such as host–pathogen interaction with human coronaviruses, interferon induction, and cholesterol biosynthesis (Daniloski et al., 2020; Sanders et al., 2021; Yoshida et al., 2022; Figure 2B and C), validating the effectiveness of the screening. Interestingly, ribosomal organization, oxidoreductase activity, and mitochondrial organization were also identified as top-regulated processes (Figure 2B and C), in agreement with our transcriptomic data (Figure 1E). We then applied more stringent filters, only considering hit genes with the gRNA score >2, and identified 525 genes, of which 44.2% were shared by three variants, 49.1% were shared by two variants, and 6.7% were only for one variant (Figure 2D, left). Importantly, the expression levels of the majority (95.3%) of candidate genes were not altered by infection with any of the variants (Figure 2D, right). These results suggest that different SARS-CoV-2 variants exploit a highly common set of host factors that are constitutively expressed and not affected by infection.

### Validation of the hits identified in the genetic screen by siRNA-mediated knockdown

**Figure 3:**
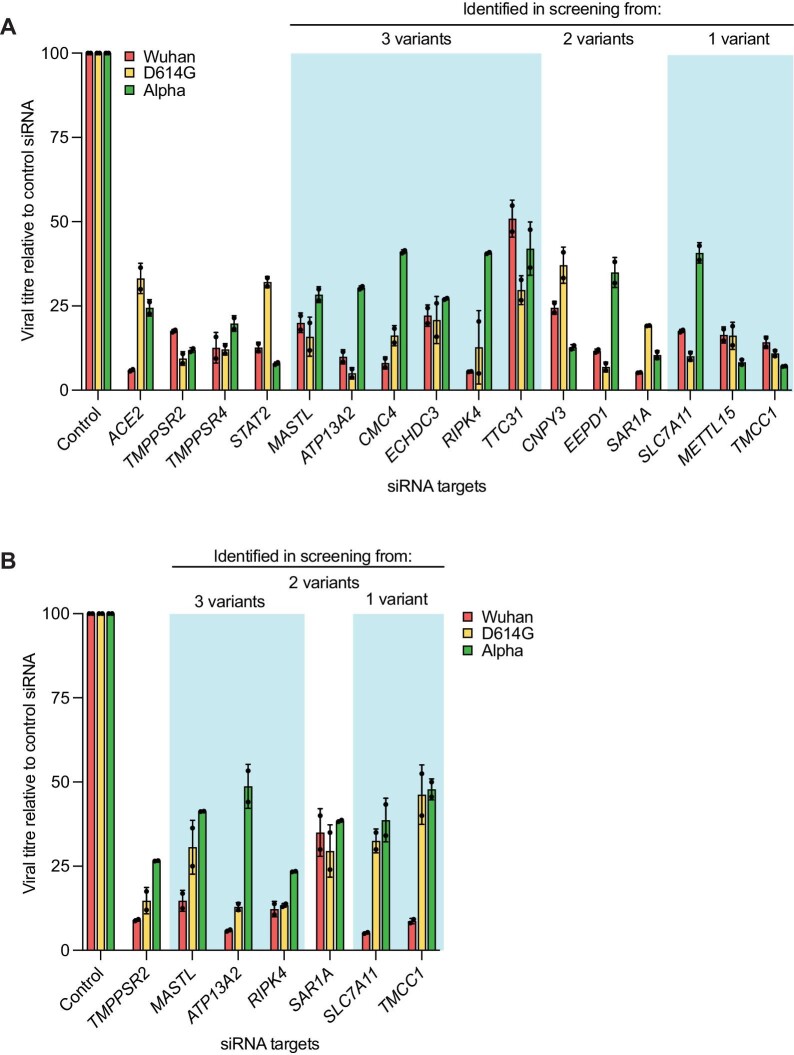
Silencing of the hit genes identified from the CRISPR-based screen reduces the production of new viral particles. Calu-3 (**A**) and Caco-2 (**B**) cells were transfected with non-targeting siRNA (control) or the indicated siRNAs and infected with SARS-CoV-2 variants (MOI = 0.1). Infective SARS-CoV-2 particles in the supernatant were assessed by PRA. The data are presented as mean ± standard deviation (SD) of two independent replicates.

We then performed secondary validation by transient siRNA-mediated knockdown. We chose siRNAs as a loss-of-function strategy because they are completely independent of CRISPR and are used in the clinic. The hits identified in our genetic screen could, in principle, act at different stages of the infection cycle, from receptor binding to virus assembly and cell exit. To monitor all viral steps in a single assay, we determined the end-point release of infective viral progeny from infected cells. We selected genes, which were common to one, two, or three variants and exhibited robust and stable expression levels during viral infection, and tested the inhibitory effect of silencing each gene on viral infection with each variant. ACE2-, TMPRSS2-, TMPRSS4-, and STAT2-silenced samples were used as positive controls, given their known role in viral entry and the interferon response (Mac Kain et al., 2022). siRNA-mediated effective hit gene knockdown (Supplementary Figure S3A) resulted in dramatic reduction in the viral titres of all three variants, to a level comparable to that of the positive controls (Figure 3A), confirming the effectiveness of our CRISPR-based screen.

It has been reported that SARS-CoV-2 also infects human gastrointestinal cells and induces gastrointestinal manifestations with consequent viral shedding in faeces (Guo et al., 2021; Krüger et al., 2021). Therefore, we further validated a subselection of hits by siRNA-mediated knockdown in the Caco-2 cell line, which is derived from the colonic epithelium and is susceptible and permissive to SARS-CoV-2 infection (Bojkova et al., 2020; Supplementary Figure S3B). As expected, knockdown of the hit genes significantly reduced the viral titres of all three variants in colon cells, indicating the conserved hits across different tissue types (Figure 3B).

### Small molecules targeting the identified hits display antiviral activity against four SARS-CoV-2 variants

Aiming to provide hits with faster clinical translation, we then searched for FDA-approved drugs and drug-like small molecules that target our hits and tested their antiviral activity. RIPK4, SLC7A11, and MASTL are three proteins that were not previously reported to be implicated in SARS-CoV-2 infection. RIPK4 interacts with protein kinase Cβ (PKCβ) and PKCδ in regulating keratinocyte differentiation, cutaneous inflammation, and cutaneous wound repair (Xu et al., 2020). RIPK4 is inhibited by Tamatinib and Vandetanib (Ton et al., 2013; Bryan and Rajapaksa, 2018). SLC7A11, also known as xCT, is the major subunit of the cystine/glutamate antiporter. SLC7A11 has been extensively studied for its role in regulating the redox state of cells, cell homeostasis, and the pathophysiology of several diseases (Jiang et al., 2015; Lim et al., 2019; Jyotsana et al., 2022). In viruses, SLC7A11 has been shown to mediate entry and post-entry events of Kaposi's sarcoma-associated herpesvirus (Kaleeba and Berger, 2006; Veettil et al., 2008; Chakraborty et al., 2012). SLC7A11 is inhibited by Sulfasalazine and imidazole ketone erastin (IKE) (Hou et al., 2020; Zhou et al., 2021). MASTL regulates mitosis and meiosis and is considered a promising anticancer target. A novel compound, called MKI-1, has recently been described as a specific inhibitor of MASTL (Kim et al., 2020).

**Figure 4:**
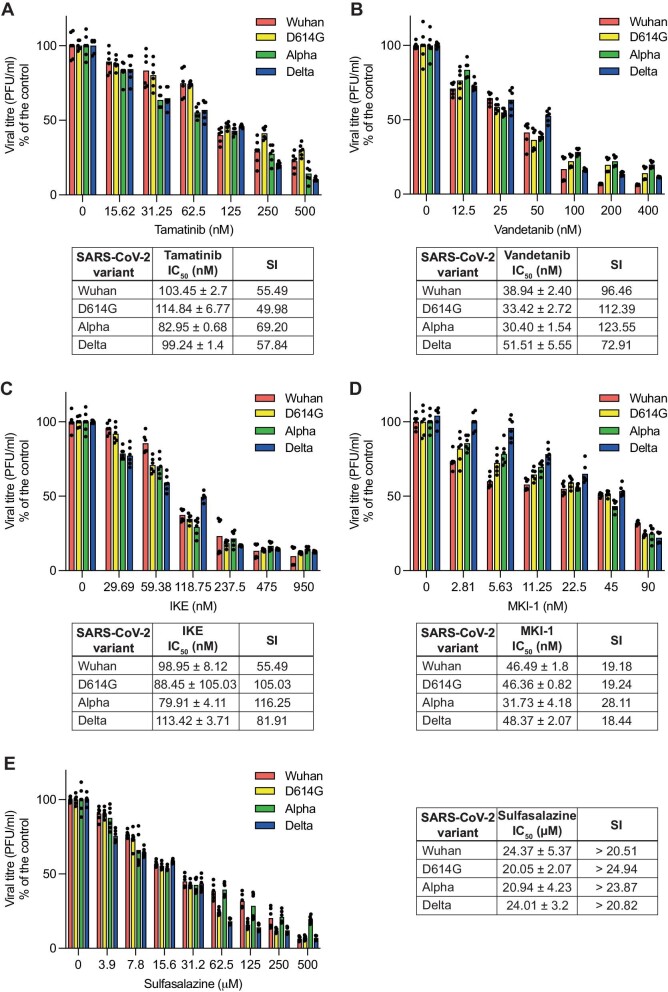
Compounds targeting the identified hits display antiviral activity in human lung cells. Calu-3 cells were pretreated with the indicated compounds for 24 h and infected with SARS-CoV-2 variants (MOI = 0.1). The compounds were added to fresh medium at 1 h.p.i. and remained in the medium throughout the experiment. At 48 h.p.i., the cell medium was subjected to PRA, and the viral titre was calculated and expressed as PFU/ml. The bars indicate the means of two biological replicates. Each condition was tested in triplicate per replicate. Individual technical replicates are shown as dots.

First, all the compounds were tested in Calu-3 cells for their cytotoxic activity, which was expressed as half maximal cytotoxic concentration (CC_50_). Most of the compounds were cytotoxic in the micromolar range (Tamatinib, Vandetanib, and IKE), MKI-1 was cytotoxic in the high nanomolar range, whereas Sulfasalazine displayed no cytotoxicity at any of the tested concentrations (Supplementary Table S3 and Figure S4). Then, the compounds were tested for antiviral activity, which was expressed as half maximal inhibitory concentration (IC_50_) with a selectivity index (SI) determined as the ratio of IC_50_ to IC_50_, at concentrations not causing cytotoxicity to host cells to avoid confounding effects. Due to the emergence and spread of the Delta variant during the course of this study (Mlcochova et al., 2021), the antiviral activity was tested against four variants (Wuhan, D614G, Alpha, and Delta). All the tested compounds exhibited potent, dose-dependent, and selective antiviral activity, as determined by the inhibition of viral progeny production at 48 h.p.i. (Figure 4). Tamatinib, Vandetanib, IKE, and MKI-1 were all highly active in the nanomolar range, inhibiting virus production by 85%–93% at the highest concentration. IKE and Vandetanib both had excellent average SIs (∼100; Figure 4B and C). Sulfasalazine induced a strong antiviral effect in the low micromolar range (IC_50_ 5.7 μM) and maintained an acceptable SI (>20) due to its negligible cytotoxicity (Figure 4E). Notably, all the tested compounds exhibited antiviral activity against the Delta variant, further supporting the ‘variant-wide’ relevance of our hit genes. Importantly, three compounds are FDA-approved drugs and are therefore suitable for drug repurposing studies. Thus, RIPK4, SLC7A11, and MASTL emerge as *bona fide* therapeutic targets for multiple SARS-CoV-2 variants.

### SARS-CoV-2 induces ROS in infected cells

Our genome-wide screen identified mitochondrial and oxidoreductive activities as critical components of the cellular response to SARS-CoV-2 infection, suggesting a potential role for ROS. Indeed, SLC7A11 is a key regulator of the cell redox state. Therefore, we investigated whether the antiviral mechanism of SLC7A11 inhibitors relies on intracellular ROS modulation. Because several independent studies have reported a higher risk of death for COVID-19 patients treated with Sulfasalazine, possibly associated with SLC7A11-independent inhibition of type I interferon production *in vivo* (Konig et al., 2022), we selected IKE to inhibit SLC7A11 in the following study.

**Figure 5:**
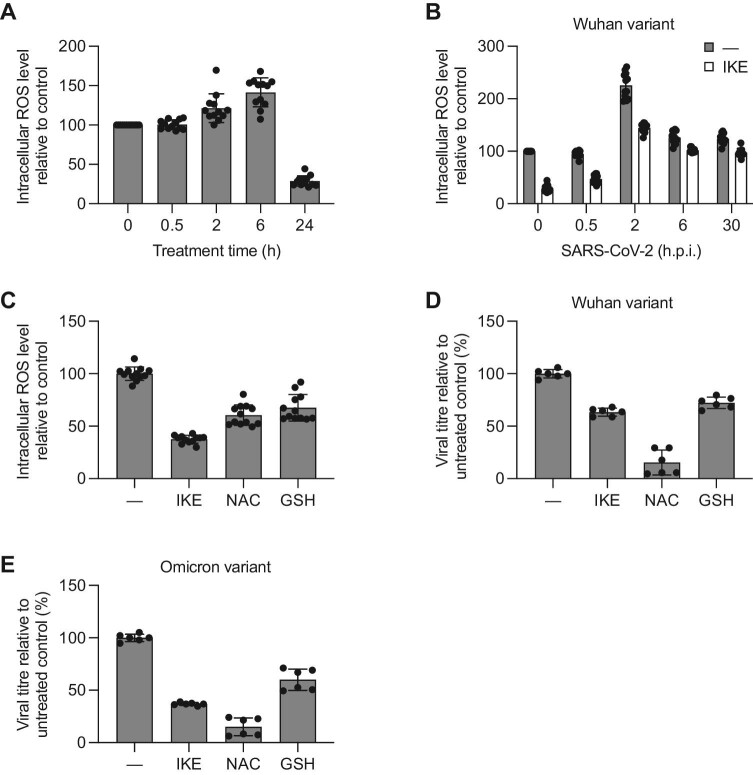
Decreased ROS levels impair viral replication. (**A**–**C**) ROS levels in Calu-3 cells were measured via the H_2_DCFDA assay. Each condition was tested in six replicates. (**A**) Intracellular ROS levels were measured at different time points after IKE administration (950 nM). (**B**) Intracellular ROS levels were measured at different time points after SARS-CoV-2 infection in the absence or presence of IKE (950 nM). In the latter case, the cells were pretreated with IKE for 24 h prior to SARS-CoV-2 infection. (**C**) Intracellular ROS levels were measured after treatment with IKE (950 nM), NAC (5 mM), or GSH (300 μM) for 24 h. (**D** and **E**) Calu-3 cells were pretreated with IKE (950 nM), NAC (5 mM), or GSH (300 μM) for 24 h prior to SARS-CoV-2 infection. Viral titres of the Wuhan (**D**) and Omicron (**E**) variants were assessed in triplicate by PRA after a single cycle of replication, i.e. 30 h.p.i. The data were normalized to the untreated control and are presented as mean ± SD of two biological replicates. Individual technical replicates are shown as dots.

We assessed intracellular ROS levels in Calu-3 cells treated with IKE for 24 h and observed a biphasic trend, with an initial increase at early time points (0–6 h) followed by a substantial decrease at a later time point (24 h) (Figure 5A). This finding is consistent with the secondary activation of compensatory mechanisms previously reported following long-term inhibition of SLC7A11 with erastin and IKE (Zhang et al., 2019, 2021). We then assessed intracellular ROS levels upon SARS-CoV-2 infection at different time points post infection and within the first replication cycle (Zhou et al., 2021; Mautner et al., 2022). Under our experimental conditions, an evident increase in ROS level was observed at 2 h.p.i., followed by a drop to the baseline level at later time points (Figure 5B). These results support the evidence that intracellular inflammation is induced by the binding of the Spike protein to the ACE2 receptor, which ultimately leads to the expression of viral non-structural proteins (Li et al., 2021; Sun et al., 2022). Pretreatment with IKE for 24 h prior to viral infection (Felgenhauer et al., 2020; Riva et al., 2020; Mukherjee et al., 2021; Narayanan et al., 2022; Teixeira et al., 2022) reduced the basal ROS level (Figure 5B, time 0 h) and kept lower ROS levels at early time points (Figure 5B, time 0.5–2 h). At later time points, ROS levels in IKE-treated cells were comparable to those in untreated cells. Thus, IKE pretreatment of infected cells prevented virus-induced ROS bursts (up to 2 h.p.i.).

We hypothesized that the increased intracellular ROS contribute to the progression of the viral life cycle, which is in line with the observation that other viruses induce ROS production for their own benefit (Foo et al., 2022). To test this hypothesis, we measured the generation of new infective virions during the first viral cycle in cells treated with IKE or two potent antioxidant molecules approved for clinical use, namely glutathione (GSH) and N-acetyl cysteine (NAC). Consistent with their antioxidant activity, GSH or NAC treatment reduced ROS levels (Figure 5C). All the treatments showed strong antiviral activity (Figure 5D) after a single replication cycle. Given the reported antiviral properties of NAC against other human pathogens (Geiler et al., 2010; Sreekanth et al., 2019), we further investigated its antiviral activity against SARS-CoV-2 in Calu-3 cells and confirmed the potent antiviral activity without cytotoxicity at the concentrations tested (CC_50 _> 5 mM, IC_50_ 0.549 mM, SI > 9.1) (Supplementary Figure S5A and B). Next, we assessed the effect of NAC on viral entry using a pseudotyped vesicular stomatitis virus (VSV) that contains SARS-CoV-2 Spike protein on its surface (Whitt, 2010; Cegolon et al., 2021; Caputo et al., 2022). Under the same conditions for the antiviral assay, we did not observe any effect of NAC on SARS-CoV-2 entry (Supplementary Figure S5C), suggesting that elevated ROS levels are critical for post-entry viral steps. Therefore, interfering with ROS levels may provide a new valuable therapeutic approach against SARS-CoV-2 infection.

While the manuscript was being prepared, the new variant of concern Omicron (BA.1) emerged and became dominant worldwide. As shown in Figure 5E, IKE, GSH, and NAC treatments were also effective against the Omicron variant, indicating that our compounds target core processes shared by previous and emerging variants.

### Treatment with NAC inhibits SARS-CoV-2 infection in vivo

**Figure 6:**
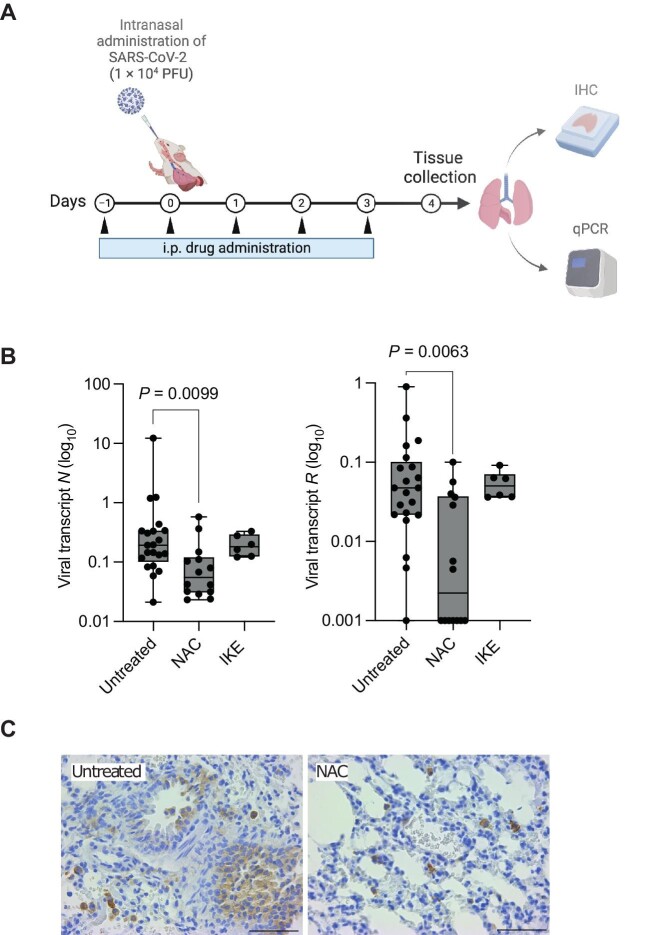
Treatment with NAC inhibits SARS-CoV-2 infection *in vivo*. (**A**) Schematic of the *in vivo* experiment created with Biorender.com. i.p., intraperitoneal injection. (**B**) qPCR analysis of viral transcripts *N* and *RdRp* (*R*) in lungs infected with SARS-CoV-2 and treated with NAC or IKE. *n* = 21 (untreated), 14 (NAC), and 6 (IKE) from two independent experiments. The data are presented as whisker plots: midline indicates the median; box indicates the 25th–75th percentile; whisker indicates minimum to maximum values. The Kruskall–Wallis corrected Dunn's test for multiple comparisons was used. (**C**) Representative images of IHC for the viral N protein. The quantification result is shown in Supplementary Figure S5D. Scale bar, 100 μm.

We then tested the effects of IKE and NAC *in vivo* using keratin 18 (K18)-hACE2 mice, which express human ACE2 receptor under the K18 promoter in the epithelia, including airway epithelial cells, and recapitulate several aspects of severe and non-severe COVID-19 in humans (Dong et al., 2022). One day after the first dose (pretreatment) of IKE or NAC, the mice were infected intranasally with the SARS-CoV-2 Delta strain and subsequently treated with IKE or NAC daily for additional four days. Then, the lungs were harvested for total RNA purification, and the viral load was determined by measuring the expression levels of two viral transcripts, nucleocapsid (*N*) and RNA-dependent RNA polymerase (*RdRp*) (Figure 6A). Remarkably, NAC-treated mice had significantly lower levels of viral transcripts than untreated mice (Figure 6B), which was confirmed by immunohistochemistry (IHC) analysis of the N protein in the lungs (Figure 6C; Supplementary Figure S5D). Thus, NAC is an effective treatment against SARS-CoV-2 infection in both human cells and a humanized mouse model of COVID-19.

## Discussion

As obligate intracellular parasites, viruses are highly dependent on their host cells: they have evolved to exploit cells for their own purposes by hijacking cellular pathways and to evade the innate immune response by modulating host factors and signalling pathways. RNA viruses, such as SARS-CoV-2, are even more dependent on host cells (Nagy and Pogany, 2012). However, current therapeutic interventions for COVID-19 only target viral proteins, which promotes the emergence of variants that evade vaccine-induced immunity or resistance to antiviral drugs. For example, the drug Paxlovid contains nirmatrelvir, an inhibitor of the major SARS-CoV-2 protease 3CL^pro^ (Owen et al., 2021; Schultz et al., 2022). Unfortunately, treatment with nirmatrelvir is associated with the emergence of naturally occurring resistant mutants (Hu et al., 2023; Iketani et al., 2023).

The aim of this study is to understand whether targeting host proteins could be an effective and safe strategy against COVID-19, as host genes are not under selective pressure. We began by asking whether different SARS-CoV-2 variants elicit similar cellular responses upon infection. The three major SARS-CoV-2 variants of concern used in our study exhibited different replication patterns in human lung cells, with the Wuhan variant producing fewer transcripts and higher titres of infective viral progeny than the D614G and Alpha variants. We attributed the differences to the reported mutations in the Spike protein. These mutations may modulate the virus–receptor binding affinity, with consequent variations in viral entry (Harvey et al., 2021). In addition, both D614G and Alpha variants harbour mutations and deletions in open reading frame 8 (Supplementary Table S1), which inhibit the cell interferon-mediated immune response (Flower et al., 2021; Rashid et al., 2021; de Souza et al., 2022), possibly explaining the enhanced RNA transcription of these two variants (Sadler and Williams, 2008; Yang and Li, 2020). However, analyses of the host transcriptome revealed qualitatively very similar transcriptional responses of the three variants, with differences only in kinetics and magnitude, suggesting that the different variants induce similar cellular responses upon infection.

We then used a genome-wide CRISPR knockout approach to gain deep insight into the host genes exploited by different variants, asking whether some genes are specifically required for one or more variants during infection. This type of approach has been successfully developed to identify the host factors exploited by other viruses (Puschnik et al., 2017; Han et al., 2018; Song et al., 2021; Grodzki et al., 2022; Zhang et al., 2022) and by SARS-CoV-2 itself (Daniloski et al., 2020; Baggen et al., 2021; Schneider et al., 2021; Wang et al., 2021b; Wei et al., 2021; Zhu et al., 2021; Kousathanas et al., 2022). However, analysis of the available data from previous SARS-CoV-2 CRISPR knockout screens did not lead to conclusions about whether different variants exploit different host factors, because different studies used different combinations of variants, cell lines, and CRISPR libraries. For these reasons, we performed a genetic screen directly comparing three variants under identical conditions, looking for the host factors that are conservatively used by all variants or required by specific variants. The rationale of our approach is twofold: (i) if a host factor is shared by all variants, it is more likely to belong to a ‘core’ of host factors essential for viral infection, and (ii) the shared host factors are more likely to be required by new variants of SARS-CoV-2 that will emerge in the future and thus may serve as a better and more comprehensive therapeutic target. Using conditions that ensured high coverage and stringency, we retrieved 525 genes whose knockout allowed cell survival during infection; 93.3% were shared by at least 2 out of 3 variants. Very satisfactorily, all the candidate genes selected by the CRISPR knockout screen were also confirmed by transient silencing of these genes. Importantly, inhibition of each tested candidate gene resulted in dramatic reduction in the viral titres of all tested variants. We conclude that the host factors that are essential for infection are entirely shared among SARS-CoV-2 variants.

We believe that the knowledge gained from this study will be instrumental in the development of host-directed therapies to control SARS-CoV-2 infection. The candidate genes we identified encode host factors, and inhibition of host factors may be a better antiviral strategy to avoid resistance. To further validate our hits and provide ready-to-trial drugs capable of stopping viral infection/replication of current and future variants, we screened a number of FDA-approved drugs for unrelated diseases and chemical compounds reported to interfere with the most common viral host factor candidates (SLC7A11, RIPK4, and MASTL). The five tested compounds displayed noteworthy antiviral activity not only against the three initially evaluated variants but also against the Delta variant, which emerged in late 2021, resulted in alarming rates of hospitalization among infected individuals of all ages, regardless of their immunization status, and was correlated with a high mortality rate (Bast et al., 2021; Sigal et al., 2022). The successful antiviral activity of the tested compounds further reinforces the power of our screening and indicates that the selected hits are crucial host factors for both the early and latest variants. Notably, these compounds showed similar efficacies against multiple variants (Figure 4), unlike previously identified inhibitors that showed reduced efficacy against new variants (Boytz et al., 2023).

We validated the hit genes identified from the CRISPR-based screen in two human cell lines derived from the lung and intestinal epithelia. Several *in vitro* human 3D systems have been developed for studying SARS-CoV-2 infection (de Melo et al., 2021; Giobbe et al., 2021; Harb et al., 2022), and it will be interesting to use these systems in the future to identify potential differences between humans and mice. However, *in vitro* models cannot take into account the complexity of virus spread into the body or the multiorgan effect of SARS-CoV-2. Therefore, we also validated *in vitro* results in a humanized mouse model.

The mechanism of action of IKE, one of the most promising compounds tested, was investigated to further validate its target, SLC7A11, against SARS-CoV-2. The central role of SLC7A11 in maintaining intracellular ROS homeostasis and its relevance as a host factor in various human viral infections have been reported (Kaleeba and Berger, 2006; Hémon et al., 2020; Liu et al., 2021; Rabinowitz et al., 2021; Yuan et al., 2022). IKE has been given proposed to neutralize SLC7A11-mediated cystine uptake and ROS modulation (Koppula et al., 2018). While elevated intracellular ROS levels trigger innate immunity-mediated antiviral mechanisms, viral infection counterintuitively stimulates ROS production, and increased ROS levels support viral propagation (Chen et al., 2020; Foo et al., 2022). Indeed, our gene expression analysis suggests reduced oxidative phosphorylation in infected cells, possibly as an attempt by the cells to reduce ROS and create a hostile environment for viral replication. We have shown that SARS-CoV-2 stimulates ROS production in human bronchial cells during the early stages of infection. The reduction in ROS levels induced by prolonged IKE administration and GSH or NAC treatment impaired the SARS-CoV-2 viral cycle. The effect of NAC treatment on COVID-19 patients has been investigated in several retrospective studies, the results of which are suggestive but not definitive (Wong et al., 2021; Faverio et al., 2022; Izquierdo et al., 2022). The mechanistic explanation has been given that the antioxidant, anti-inflammatory, and anti-thrombotic effects of NAC counteract viral pneumonia; however, results from ongoing randomized controlled trials are needed to draw firm conclusions (Faverio et al., 2022). It is also important to note that targeting host factors may reduce the development of resistance, although side effects are a major concern. Therefore, combination therapy of compounds targeting host factors with direct-acting antivirals may be a more effective approach. In conclusion, our results demonstrate the direct antiviral effects of NAC on lung epithelial cells in addition to its immunomodulatory effects. We therefore support the use of NAC and other antioxidant drugs as safe and accessible anti-SARS-CoV-2 therapies against current and future variants.

## Materials and methods

### Compounds

The tested compounds IKE (Cayman Chemical, 27088), MKI-1 (ChemBridge, 9335496), Sulfasalazine (MedChemExpress at MedChemTronica EU, HY-14655), Tamatinib (Merck Life Science, 574714), and Vandetanib (Merck Life Science, SLM2983) were dissolved in dimethyl sulfoxide (DMSO) and stored in aliquots at −20°C until use. NAC (Sigma–Aldrich, A9165) was reconstituted in sterile water, and the pH was adjusted to 7–7.4 with sodium hydroxide prior to use.

### Cell culture and virus

Vero E6 (ATCC, CRL-1586) cells were maintained in Dulbecco's modified Eagle medium (DMEM; Thermo Fisher Scientific). Calu-3 cells (ATCC, HTB-55) and Caco-2 cells (a kind gift from Prof. Stefano Piccolo at University of Padua) were maintained in DMEM/F-12 (Thermo Fisher Scientific). Both culture media were supplemented with 10% (*v*/*v*) fetal bovine serum (FBS; Thermo Fisher Scientific) and penicillin/streptavidin (Thermo Fisher Scientific). Cell cultures were maintained at 37°C and 5% CO_2_ in a humidified atmosphere and routinely tested for mycoplasma contamination (Euroclone, EMK090020). For seeding and subcultivation, the cells were first washed with phosphate-buffered saline and then incubated in the trypsin/EDTA solution (Gibco, Thermo Fisher Scientific) until the cells were detached. After the cells were seeded, the ROCK inhibitor Y27632 (10 μM; Axon MedChem, #1683) was added to the culture medium and the cells were incubated for 24 h.

The SARS-CoV-2 Wuhan isolate (SARS-CoV-2/human/ITA/CLIMVIB2/2020) was provided by the Virology Unit of Luigi Sacco University Hospital (GenBank accession ON062195 MW000351.1). The SARS-CoV-2 D614G isolate (SARS-CoV-2/human/USA/USA-WA1/2020) was provided by The University of Texas Medical Branch (GenBank accession MT576563.1). The SARS-CoV-2 Alpha and Delta isolates (human nCoV19 isolate/England/MIG457/2020 and hCoV-19/Netherlands/NH-RIVM-27142/2021_P2) were supplied by the European Virus Archive GLOBAL (EVA-GLOBAL) platform. The SARS-CoV-2 Omicron variant was provided by the Microbiology Unit of Padua University Hospital as previously described (GenBank accession ON062195; Lavezzo et al., 2022). All viral stocks were prepared by propagation in Vero E6 cells in DMEM supplemented with 2% FBS. The viral titre was assessed by plaque reduction assays (PRA) and expressed as plaque forming units (PFU) per milliliter (ml). All experiments involving live SARS-CoV-2 were performed in compliance with the Italian Ministry of Health Guidelines for Biosafety Level 3 (BSL-3) Containment Procedures in the approved laboratories of the Molecular Medicine Department of University of Padua.

### CRISPR-based loss-of-function screen

Calu-3 cells were transduced with a lentiviral vector expressing Cas9 together with the GeCKO v2 library (Creative Biogene, CCLV0001). We used an MOI of 0.3 and a coverage of 500×.

Transduced cells were selected with 4 μg/ml puromycin and cultured for at least two weeks. In parallel, the cells were infected with three different SARS-CoV-2 variants at an MOI of 3. Scattered clonal populations of the cells that survived after SARS-CoV-2 infection were expanded, lysed, and pooled together. Genomic DNA was purified with phenol/chloroform using Ampure XP (Beckman, A63881) and used for PCR amplification. The PCR products were purified using Ampure XP beads and run on 2% E-Gel™ EX Agarose Gels (Thermo, G401002) to select a band of ∼250 bp. The obtained DNA was used for library preparation with an NEBNext^®^ Ultra™ DNA Library Prep Kit for Illumina^®^ (NEB, E7370L). Libraries were run on NovaSeq 6000 (Illumina) using the NovaSeq 6000 SP Reagent Kit v1.5 (100 cycles; Illumina, 20028401). Data processing was conducted using MAGeCK software (Li et al., 2014) in combination with a custom pipeline. The gRNA score that represents the number of biological replicates in which a gRNA for a given gene is enriched in infected cells compared to uninfected cells was calculated for each variant (0–3 replicates) or combined across all variants (0–9 replicates). Genes targeted by at least two independent gRNAs or with >1000 counts in at least one sample were initially considered as screen hits. To increase the stringency, we then only considered genes with a total gRNA score >2. We also calculated average expression levels of these genes in Calu-3 cells and filtered out genes with <30 normalized counts. Additional details are provided in Supplementary Materials and methods.

### Cytotoxicity evaluation of the tested compounds

The cytotoxicity of the tested compounds was assessed and expressed as CC_50_. Calu-3 cells (2.75 × 10^4^ cells/well) were seeded in 96-well plates, and the tested compounds or an equal volume of vehicle (DMSO) were added to the medium. After 48 h, cell viability was determined by measuring the adenosine triphosphate (ATP) content of the cells using the ATPlite kit (PerkinElmer, 6016941) according to the manufacturer's instructions. CC_50_ values were calculated using the Reed and Muench method (Ramakrishnan, 2016).

### Antiviral assays

Calu-3 cells (2.75 × 10^4^ cells/well) were seeded in 96-well plates, and the tested compounds or an equal volume of vehicle (DMSO) were added to the medium. After 24 h, cell culture medium was removed and replaced with virus inoculum (MOI of 0.1). After 1 h of incubation at 37°C, the virus inoculum was removed and replaced with fresh DMEM/F-12 supplemented with 10% FBS and the tested compounds or the vehicle. The cells were incubated at 37°C for 48 h before the supernatants were harvested. The viral titre (expressed as PFU/ml) was calculated by PRA in Vero E6 cells. IC_50_ values were calculated using the Reed and Muench method (Ramakrishnan, 2016).

### Data availability

The raw sequencing data (RNAseq and CRISPR screen) have been deposited in GEO (accession number: GSE207981). The custom code generated for RNAseq and CRISPR screening analyses is available upon request.

## Supplementary Material

mjae004_Supplemental_Files

## Notes

**Author contributions:** S.N.R., M.M., and G.M. conceived the study. I.F. and C.S. performed the BSL-3 experiments. L.D. performed computational analyses. M.Z., E.C., and M.P.D. performed the cell culture and molecular biology experiments. L.V. and D.C. performed the NGS experiments. A.D.P. and A.P. performed the BSL-3 experiments with humanized mouse models. R.V. and F.T. performed histopathological analyses. A.R., S.N.R., M.M., and G.M. provided overall supervision and secured grants. M.M., L.D., and I.F. prepared the figures. I.F., S.N.R., M.M., and G.M. wrote the manuscript with help from all the authors.
