# Supplementary material for: Identification of druggable host dependency factors shared by multiple SARS-CoV-2 variants of concern
Source: J Mol Cell Biol. 2024 Feb 1;16(3):mjae004. doi: 10.1093/jmcb/mjae004 (PMC11411213; doi:10.1093/jmcb/mjae004)
Supplement: mjae004_Supplemental_Files [file mjae004_supplemental_files.zip › Supplementary Table S1.pdf]

Protein/strain	Wuhan	D614G	Apha	Delta
ORF1AB (7096 aa)<br>*same aa residue			A1707D<br>A2123V<br>Del 3673-3675 LSG<br>F3676*L<br>N4355K<br>K5781R<br>E6282G	Del 141-143 KSF<br>P306*L (P309)<br>P1637*L (P1640)<br>D2878*N<br>F3135*S<br>H3577*Q<br>K6708R
Orf1ab leader prot (nsp1)				Del 141-143 KSF
nsp2				
nsp3 (IT from MT seq)				
nsp4 (IT from MT seq)				D217N<br>F375S
nsp3C-like protease				
nsp6			Del 102-105 LSG<br>F105L	H11Q
nsp7				
Nsp8				
Nsp9				
Nsp10				
Nsp11 (not considered a viral protein any longer)				
RNAdepRNApol (nsp12)		L623P		
Helicase (nsp13)			K460R	
3'-5' Exonuclaese (nsp14)			E347G	
Endoribonuclease (nsp15)				K259R
2'-O-ribose methyltransferase (nsp16)				
S		D614G	Del69-70 HV<br>Del145Y<br>N498Y<br>A567D<br>P678H<br>T713I<br>D1115H	T19R<br>K77T<br>G142D<br>Del156-57 EF<br>L452R<br>T476K<br>P679R
E				
M				I82T
ORF6				
ORF7a				V82A
ORF7b				
ORF8		L84S	Truncated at 26th AA<br>(nt 27956 stop codon)	
N			D3L<br>R203K<br>G204R	D63G<br>R203M<br>S235F<br>D377Y
ORF10				

Supplementary Table 1. Amino acidic mutations present in the SARS-CoV-2 variants used in this study. Only mutated positions are reported. The Wuhan variant was used as reference strain, as it represented the first circulating SARS-CoV-2 isolate. Each mutation (such as A1707D) is indicated by a first letter that is the symbol for the reference amino acid on the reference Wuhan variant (e.g. A), a number for the amino acid position in the analyzed variant, and a second letter representing the amino acid actually found in the analyzed sequence (e.g., D).
