# Supplementary material for: Identification of druggable host dependency factors shared by multiple SARS-CoV-2 variants of concern
Source: J Mol Cell Biol. 2024 Feb 1;16(3):mjae004. doi: 10.1093/jmcb/mjae004 (PMC11411213; doi:10.1093/jmcb/mjae004)
Supplement: mjae004_Supplemental_Files [file mjae004_supplemental_files.zip › Supplementary Table S3.pdf]

Compound	CC_{50} (μM)
Tamatinib	5.740 ± 0.354
Vandetanib	3.756 ± 0.132
Sulfasalazine	> 500
IKE	9.290 ± 0.324
MKI-1	0.892 ± 0.001

Supplementary Table 3. The cytotoxicity (CC50) of the the used drugs was determined in Calu-3 cells. Data are mean ± s.d. of n = 2 biological replicates. Each biological replicate included three technical replicates.
