# Supplementary material for: Identification of druggable host dependency factors shared by multiple SARS-CoV-2 variants of concern
Source: J Mol Cell Biol. 2024 Feb 1;16(3):mjae004. doi: 10.1093/jmcb/mjae004 (PMC11411213; doi:10.1093/jmcb/mjae004)
Supplement: mjae004_Supplemental_Files [file mjae004_supplemental_files.zip › Supplementary Table S4.pdf]

Supplementary Table 4.: Sequence of primers used in this study.

Sars-CoV-2 ORF1ab R	for	GTGAAATGGTCATGTGTGGCG
	rev	GCATAAGCAGTTGTGGCATCTCCT
Sars-CoV-2 N	for	CACATTGGCACCCGCAATC
	rev	TGGCAATGTTGTTCCTTGAGGAAGT
human ATF3	for	ATCACAAAAGCCGAGGTAGC
	rev	GCACTCCGTCTTCTCCTTCTT
human EGR1	for	ACCCCTCTGTCTACTATTAAGGC
	rev	TGGGACTGGTAGCTGGTATTG
human GAPDH	for	CGAGATCCCTCCAAAATCAA
	rev	GGCAGAGATGATGACCCTTT
human TMPRSS2	for	CAAGTGCTCCAACTCTGGGAT
	rev	AACACACCGATTCTCGTCCTC
human TMPRSS4	for	CCAAGGACCGATCCACACT
	rev	GTGAAGTTGTCGAAACAGGCA
human ACE2	for	TCCGTCTGAATGACAACAGC
	rev	TCACTCCCATCACAACTCCA
human RIPK4	for	CTAGCTGCCGTTCGTTTCTC
	rev	ACCTCTTCCCCAAGACTGGT
human ECHDC3	for	AGTGGAGGCAGAGGAGTGAG
	rev	AGGCCAAATGCTATGACACC
human CMC4	for	GCTGTTCTCAAGGCGGATTA
	rev	TCCATGTAGCTGTTGGCTTG
human MASTL	for	TGATACGGTTTTGCCACCTT
	rev	ATTACAGGCATGAGCCATCG
human METTL15	for	CCAGTGTGAGCAACAGAACG
	rev	CAATCTCTGCCTCCAGCTTC
human SAR1A	for	CACTCCCCGACATACTCGTT
	rev	CTTTGCTCACAGGCCATACA
human ATP13A2	for	GCAGAAGCCTCAGTGGTCTC
	rev	GACGCTGAACGAAGTGTCAA
human TTC31	for	CTGGGCCTTTCTCTTCCTCT
	rev	TAATCTGGGGGCTATGGCTA
human EEPD1	for	AATGGGATGTCTGAGCCTTG
	rev	TGCAAAAAGCAATCTGGCTA
human TMCC1	for	GAAACGTCCCCTCATTTGAA
	rev	AGCTTCACTCACCCAGGCTA
human CNPY3	for	AGCAGCAAACAAAGGAAGGA
	rev	TCAAAATCAGGGGTCTCAGG
human STAT2	for	CCTCCCCCATAAAATGATCC
	rev	AAATCCCCAGCAATCCTACC
human SLC7A11	for	TATCCCTGGCATTTGGACGC
	rev	AGAAAATCTGGATCCGGGCG
