# Supplementary material for: Identification of druggable host dependency factors shared by multiple SARS-CoV-2 variants of concern
Source: J Mol Cell Biol. 2024 Feb 1;16(3):mjae004. doi: 10.1093/jmcb/mjae004 (PMC11411213; doi:10.1093/jmcb/mjae004)
Supplement: mjae004_Supplemental_Files [file mjae004_supplemental_files.zip › [01]SUPPLEMENTARY INFORMATION_Methods and figures.pdf]

SUPPLEMENTARY INFORMATION

Identification of druggable host dependency factors shared by multiple SARS-CoV-2 variants of concern

Ilaria Frasson, Linda Diamante, Manuela Zangrossi, Elena Carbognin, Anna Dalla Pietà, Alessandro Penna, Antonio Rosato, Ranieri Verin, Filippo Torrigiani, Cristiano Salata, Marìa Paula Dizanzo, Lorenzo Vaccaro, Davide Cacchiarelli, Sara N. Richter, Marco Montagner, Graziano Martello

Inventory of Supplementary information:

Supplementary Materials and Methods
5 Supplementary Figures

Supplementary Materials and Methods

SARS-CoV-2 titration by plaque reduction assay

Vero E6 cells were seeded in 24-well plates at a concentration of $9x10^4$ cells/well. The following day, serial dilutions of the viral stocks or tested supernatants were performed in serum-free DMEM media. After 1 h absorption at 37 °C, 2x overlay media was added to the inoculum to give a final concentration of 2% (v/v) FBS/DMEM media and 0.6% (v/v) methylcellulose (Merck Life science, Cat: M0512) to achieve a semi-solid overlay. Plaque assays were incubated at 37 °C for 48 h. Samples were fixed using 5% Formaldehyde in PBS (Merck Life Science, Cat: 252549) and plaques were visualised using Crystal Violet solution (20% Ethanol, Merck Life science, Cat: C6158).

Virus infections in human lung cells and RNA sequencing analysis

Calu-3 cells were seeded ($1.2x10^5$/well) in 12-well plates, and after 24 h the cell culture supernatant was removed and replaced with virus inoculum (MOI 1). Following 1 h adsorption at 37 °C, the virus inoculum was removed, the cell monolayer was washed in PBS prior to medium replacement (10% FBS DMEM/F-12). At 6, 9, 12 and 24 h post infection (h.p.i.), cells were harvested and total RNA purified with Total RNA Purification Kit (NorgenBiotek, Canada, Cat #48400), following manufacturer's protocol. Total RNA was retrotranscribed with random primer and M-MLV Reverse Transcriptase (Thermo Fisher Scientific, 28025013). qPCR analysis was carried out in a QuantStudio 6 Flex RealTime PCR System (Thermo Fisher Scientific) with FastStart SYBR Green Master mix (Roche, Cat. 04673492001). Primers for qPCR are listed in Supplementary Table 4.

RNA sequencing

Total RNA was isolated with Total RNA purification kit (Norgen Biotek, Cat #48400) and Quant Seq 3' mRNA-seq Library Prep kit (Lexogen) was used for library construction. Sequencing was performed on the Illumina NextSeq 500 instrument with a coverage of ~5 million reads (75bp SE) per sample. Raw reads obtained from RNAseq were mapped to a hybrid human (GRCh38.p13) and SARS-CoV-2 reference genome (GenBank: NC_045512.2) using STAR (v. 2.7.6a). The gene expression levels were quantified using Subread package featureCounts (v. 2.0.1). STAR parameters were set following Lexogen guidelines for data analysis (https://www.lexogen.com/quantseq-data-analysis/), while featureCounts was used with default parameters.

All RNA-seq analyses were carried out in the R environment (v. 4.1.0) with Bioconductor (v. 3.7). Genes were sorted removing those with a total number of raw counts below 10 in at least 4 samples. After applying this filter, we identified 11,880 expressed genes that were considered for further analyses. Outlier replicates were removed following quality control and

clustering analysis. Differential expression analysis was computed using the DESeq2 R package (v. 1.32.0) (Love et al., 2014). DESeq2 performs the estimation of size factors, the estimation of dispersion for each gene and fits a generalised linear model. Transcripts with absolute value of FC >1.5 (log2[FC] >0.59) and an adjusted p-value <0.05 (Benjamini-Hochberg adjustment) were considered significant and defined as differentially expressed genes (DEGs) for the comparison in analysis. Volcano plots (Figure 1B) were generated with log2[FC] and −log10[q-value] from DESeq2 differential expression analysis output using the ggscatter function from the ggpubr R package (v. 0.4.0). Heatmaps were made using DESeq2-normalised values with the pheatmap function from the pheatmap R package (v.1.0.12) on viral genes (Figure 1A) or selected markers (Figure 1C and Figure 2D). Statistics and visualisation of the correlation matrix in Figure 1D were performed with the Hmisc (v. 4.5-0) and Corrplot (v. 0.90) packages using Pearson's correlation method.
Biological significance of DEGs was explored by GO term enrichment analysis (Figure 1E, Supplementary Figure S2A and Figure 2B) using the enrichR package (v.3.0) (Chen et al., 2013).

Protein network analysis

Protein network was generated with STRING 11.5 (online tool: https://string-db.org/) and Cytoscape stringApp. A first network was generated with STRING by using all the proteins belonging to the following libraries: Wikipathways (Host-pathogen interaction of human coronaviruses - interferon induction WP4880, Type I interferon induction and signalling during SARS-CoV-2 infection WP4868), GO Biological Processes (mitochondrion organisation (GO:0007005), negative regulation of TOR signalling (GO:0032007)), GO Molecular Functions (ribosomal large subunit binding (GO:0043023), RNA binding (GO:0003723), oxidoreductase activity, acting on the aldehyde or oxo group of donors, NAD or NADP as acceptor (GO:0016620)). This network was exported to Cytoscape and manually curated to facilitate visualisation by removing unconnected nodes (score > 0.70). Edges show connections based on experiments, coexpression, text mining, databases, cooccurrence, neighbourhood, fusion (any) and edges width is proportional to the strength of the interaction (mapping type “continuous” based on “Stringdb score” value).

CRISPR-based loss-of-function screen

Calu-3 cells stably expressing SpCas9 were generated by transducing Calu-3 cells with lentivirus expressing Cas9 under the EFS promoter (provided with the library by Creative Biogene, see below) and selection with 2 µg/ml blasticidin. Transduction conditions were optimised to avoid non-specific effects of Cas9 on SARS-Cov-2 cytopathic effect. We conducted genome-wide negative selection (dropout) screens in Cas9-Calu-3 cells by using the human GeCKO v2 library (Creative Biogene, cat. CCLV0001) that targets 18823 genes with 6 gRNAs/gene as well as 1000 non-targeting gRNAs. The library is provided as two pooled DNA half-libraries (Library A and B, 3 gRNAs/gene each) that we screened in parallel. On day 1, four T175 flasks were seeded with 18.6×10^{6} Cas9-Calu-3 cells, the number of cells was optimised in order to have $15.5\text{x}10^{6}$ cells the following day. On day 2, 8.36 µl of Library A viral particles (stock 5.56×10^{8} TU/ml) and 7.92 µl of Library B viral particles (stock $5.87\text{x}10^{8}$

TU/ml) were resuspended in 20 ml medium and used to transduce two T175 flasks/semilibrary (20 ml/flask) with an MOI of 0.3. These conditions allowed a coverage of 500×, i.e. each gRNA is present, on average, in 500 unique cells and the majority of transduced cells received a single viral integrant. Transduced cells were selected with 4 µg/ml with puromycin and cultured for at least 2 weeks, after which cells transduced with the same semilibrary were pooled together. For each of the 4 conditions (infection with Wuhan, D614G and Alpha SARS-CoV-2, and a Mock sample) and each semilibrary, we plated 3 replicates, each of $6x10^6$ cells/replicate. These cells were spread into 12 wells (6-well format) to allow an even distribution of the cells (see Supplementary Figure S5 for a schematics of the screening layout).

The day after, 72 wells (36 wells for each semilibrary) were infected, in parallel, with each of the SARS-CoV-2 variants at a MOI of 3. After 48 h, we observed complete death of non-infected control cells, and appearance of scattered clonal populations of cells that survived SARS-CoV-2 infection. We expanded the colonies for 28 days in order to obtain a number of cells suitable for detection of each clone. The cell medium was changed every 48 h. Then, all the wells transduced with the same gRNA library and infected with the same SARS-CoV-2 variant were lysed, pooled together and genomic DNA purified with phenol/chloroform. We considered this as a replica. Thus, for each gRNA semilibrary and SARS-CoV-2 variant we obtained and sequenced 3 replicates.

The gRNA cassettes of the surviving clones were identified as follows: gDNA from cells was extracted through Phenol-Chloroform and purified with ethanol precipitation to obtain the maximum extraction efficiency. The obtained gDNA was then purified using AmpureXP (Beckman A63881). Purified gDNA was quantified with Qubit 1× dsDNA High Sensitivity (Thermo Q33231) and subsequently used for PCR amplification. PCR was performed using KAPA HiFi HotStart ReadyMix (Roche #7958927001) at Tm 60°C for 15 cycles. The primers used were: GECKO2_Fwd: GCTTTATATATCTTGTGGAAAGGACGAAACACC; GECKO2_Rev: CCGACTCGGTGCCACTTTTTCAA. The PCR reaction was purified using Ampure XPbeads and run on 2% E-Gel™ EX Agarose Gels (Thermo G401002) to select a band of about 250 bp. DNA from agarose gel was purified using Zymoclean Gel DNA Recovery Kit (Zymo D4007). Obtained DNA was used for library preparation with NEBNext® Ultra™ DNA Library Prep Kit for Illumina® (NEB E7370L). Libraries were run on Novaseq 6000 (Illumina) on NovaSeq 6000 SP Reagent Kit v1.5 (100 cycles) (Illumina 20028401).

Data processing was conducted using the MAGeCK software (Li et al., 2014) in combination with a custom pipeline. Briefly, read counts from different samples were first mapped to the reference gRNA sequences library using “mageck count” function with default parameters; as the sequencing library is unstranded, reads were mapped also to the reverse complement of the gRNA library and then counts were combined.

Individual gRNA-level and aggregate gene-level enrichment analysis was performed using a custom pipeline. gRNA counts from different samples were normalised to total counts to adjust for the effect of library sizes. Only gRNAs with a count number higher than the maximum count value of control samples (CTRL, cell transduced with the GeCKO library that were not infected) were considered enriched and thus retained for further analyses.

We calculated a gRNA score that represents the number of biological replicates in which a gRNA for a given gene were found enriched over control samples. The gRNA score was calculated within each variant (ranging from 0 to 3 replicates) or combining all variants (ranging from 0 to 9 replicated).

Genes were considered screen hits if targeted by at least 2 independent gRNAs and if the number of counts >1000 at least in one sample; to increase stringency of the analysis, we considered only genes with a total gRNA score >2 and we also calculated the average expression in Calu-3 cells and filtered out genes with <30 normalised counts.

Validation of candidate genes

Selected candidate genes were validated in Calu-3 and Caco-2 cell lines by transient transfection. Cell reverse transfections were carried out using HiPerFect (Qiagen, 301704) for Calu-3 (2.5×10^4 cells/well in 96-well format) and forward transfections with Lipo3000 were done (Thermofisher, L3000015) for Caco-2 cells (1×10^4 cells/well in 96-well format) . The siRNAs were selected from the FlexiTube GeneSolution 4 siRNA sets (Qiagen) and transfected as a mix at 24 nM in Calu-3 and 10 nM in Caco-2 following manufacturer's instructions. As a negative control for our transfections we used a non-targeting siRNA from Qiagen (SI03650318, sequence: UUCUCCGAACGUGUCACGU). Cells were harvested 48 h post-transfection, their total RNA was purified and retrotranscribed as in (Zangrossi et al., 2021a). Real-time PCR was performed as in Zangrossi et al. (2021b) with primers listed in Supplementary Table S4. Small interfering RNA used in this study are listed in Supplementary Table S5.

At 24 h post-transfection, the cell culture supernatant was removed and replaced with virus inoculum (MOI = 0.1). Following 1 h adsorption at 37 °C, the virus inoculum was removed and replaced with fresh 10% FBS DMEM/F-12 media. Cells were incubated at 37°C for 48 h before supernatants were harvested. The viral titre (expressed as PFU/ml) was calculated by PRA in Vero E6 cells.

ROS measurements

ROS measurement was performed by H_2DCFDA assay, according to the manufacturer's instructions (Thermo Fisher Scientific, D399). In brief, Calu-3 cells (2.75×10^4 cells/well) were seeded in 96-well plates. Tested compounds or an equal volume of vehicle (DMSO) were supplemented to the medium 24 h prior to ROS analysis, if not otherwise stated. Each condition was tested in sextuplicate. Following drug treatment, media was removed and cells were incubated with 10 µM H_2DCFDA in phenol-red free media for 20 min at 37 °C. Cells were washed with clear medium to remove free probe and fluorescence intensity (excitation=485 nm; emission=530 nm) was measured using a microtiter plate reader (Promega, GloMax Microplate reader). In each experimental plate, an additional lane of control cells was treated for 3 min at 37°C with H_2O_2 (3.6% w/v), to test probe correct fluorescence.

Pseudotyping of VSV

rVSVΔG-Luc was provided by Michael Whitt, University of Tennessee, Memphis, USA. Expression plasmids for glycoprotein (VSV-G), and SARS-CoV-2 spike protein (pCAGGS_SARS-CoV-2_spike, provided by the National Institute for Biological Standards and Control, NIBSC), used for the generation of SARS-CoV-2 pseudovirus, were previously

reported (Whitt, 2010). VSV pseudotypes were generated following published protocols (Rentsch and Zimmer, 2011). In brief, 293T, transfected with Lipofectamine 3000 (ThermoFisher Scientific, Milan, Italy, L3000015) to express the viral surface glycoprotein under study, was inoculated with a replication-deficient VSV vector that contains the expression cassette of firefly luciferase in place of the VSV-G open reading frame, VSV-ΔG-fLuc. After an incubation period of 1 h at 37°C, the inoculum was removed, cells were washed with PBS, and fresh culture medium was added to the cell monolayer. Pseudotyped particles were harvested 16 h post inoculation and clarified from cellular debris by centrifugation and used for experiments.

SARS-CoV-2 or VSV Pseudoviruses infections

Calu-3 cells were seeded in culture medium the day before infection (7×10^4) in 24-well plates and subsequently vehicle-treated or treated with NAC (5 mM). Complete culture medium (DMEM-F12 supplemented with 10% FBS) was removed, and cells were inoculated with SARS-CoV-2 pseudovirus or with the wild-type VSV-g pseudovirus (in DMEM-F12 no FBS) at a MOI of 0.05 for 1 h at 37°C in a humidified incubator. 1 h.p.i., the medium containing the pseudovirus was removed, cells were washed in 1x PBS, and fresh complete medium was added to each well. Infection efficiency was quantified 24 h.p.i. by measuring the firefly luciferase in cell lysates using a commercial substrate (Britelite plus, PerkinElmer Italia, Milan, Italy, 6066766) in a plate luminometer (Varioskan Lux, Thermofisher). Luciferase values were normalized to the cellular protein content (Pierce™ BCA Protein Assay Kit, ThermoFisher Scientific, Rodano, Milan, Italy, 23227).

Animal studies

Six- to 8-week-old B6.Cg-Tg(K18-ACE2)2Prlmn/J transgenic mice were purchased from The Jackson Laboratory and bred at the IOV-IRCCS Specific Pathogen-Free animal facility. K18-hACE2 mice were acclimatised in the BSL-3 facility for 72 h prior to treatment. Mice were treated intraperitoneally either with 150 mg/kg of NAC or 40 mg/kg of IKE, or vehicles as controls. One day after the first dose (pre-treatment), mice were infected intranasally with 10 µl of 1×10^4 PFU of SARS-CoV-2 Delta strain. Mice received drugs daily for 5 days, and were euthanized at 4 days post infection. Body weight and physiological conditions were monitored daily until sacrifice, when lungs were collected for further analysis. The whole right lobe was fixed in 10% buffered-formalin for histopathology, while the left lobes were added with Trizol (Invitrogen) for RNA extraction. All the procedures involving animals and their care were in conformity with institutional guidelines that comply with national and international laws and policies (D.L. 26/2014 and subsequent implementing circulars), and the experimental protocol (Authorization n. 355/2021-PR) was approved by the Italian Ministry of Health.

Assessment of viral transcripts *in vivo*

At 4 days post infection, lungs were harvested in 2 ml of Trizol and homogenized using a gentleMACS Octo dissociator (Miltenyi Biotec, Inc.). Total RNA was purified with

trizol/chloroform, genomic DNA digested with DNAse (DNAse I, Ambion, Thermofisher AM222) treatment followed by a second round of purification with phenol/chloroform/isoamyl alcohol. qPCR analysis was carried out in a QuantStudio 6 Flex RealTime PCR System (Thermo Fisher Scientific) with TaqPath 1-Step RT-qPCR (Applied Biosystems, Thermofisher, A15299). Primers for qPCR are listed in Supplementary Table 4. Probe for N transcript: CTAACAATGCTGCAATCGTGC (Reporter: FAM; Quencher: TAMRA). Probe for R transcript: CTATATGTTAAACCAGGTGGAACC (Reporter: FAM; Quencher: TAMRA). Probe for *ApoB* transcript: CCA ATG GTC GGG CAC TGC TCA A (Reporter: VIC; Quencher: TAMRA). Expression of viral transcripts in each sample was calculated with the formula 2^(Ct *ApoB*- Ct N/R). Statistical analysis was performed with Graphpad Prism 9 Version 9.4.1.

Immunohistochemistry of murine lung tissue

After tissue harvesting, the right lung lobe was fixed in 10% buffered-formalin, dehydrated through a graded series of ethanol and embedded in paraffin (FFPE). Immunohistochemical (IHC) examinations of 4 μm thick lung sections were performed on polarised glass slides (TOMO, Matsunami Glass IND, Osaka). Heat-induced antigen retrieval with 0.01 M Sodium citrate buffer, pH 6.0 for 60 minutes, at 97°C was followed by blocking of nonspecific bindings with 5% bovine serum albumin. Primary anti-SARS-CoV-2 nucleocapsid rabbit polyclonal antibody (Pro Sci Incorporated, Flint, CA, Cat: 9099, 1:300) was applied overnight at room temperature in a humidified chamber. Slides were then incubated with a HRP-conjugated secondary anti-rabbit antibody (Invitrogen, Carlsbad, CA, Cat: 31460, 1:500) for 60 minutes at room temperature. After endogenous peroxidase blocking (Agilent Technologies, Santa Clara, CA, Cat: S2023), 3,3'-diaminobenzidine peroxidase substrate detection kit (Agilent Technologies, Santa Clara, CA, Cat: K3467) was used to detect immunoreactivity. Non-infected murine pulmonary tissue was used for negative controls. Intensity of signal was subjectively scored in different anatomical compartments (i.e. blood vessels, interstitium, an airways/alveoli) as follows: 0, not detected; 1, mild/weak; 2, moderate; 3, strong. Nucleocapsid protein is detected primarily in alveolar pneumocytes type II and interstitial macrophages. Finally, a total IHC cumulative score for each section was obtained.

SUPPLEMENTARY FIGURE 1

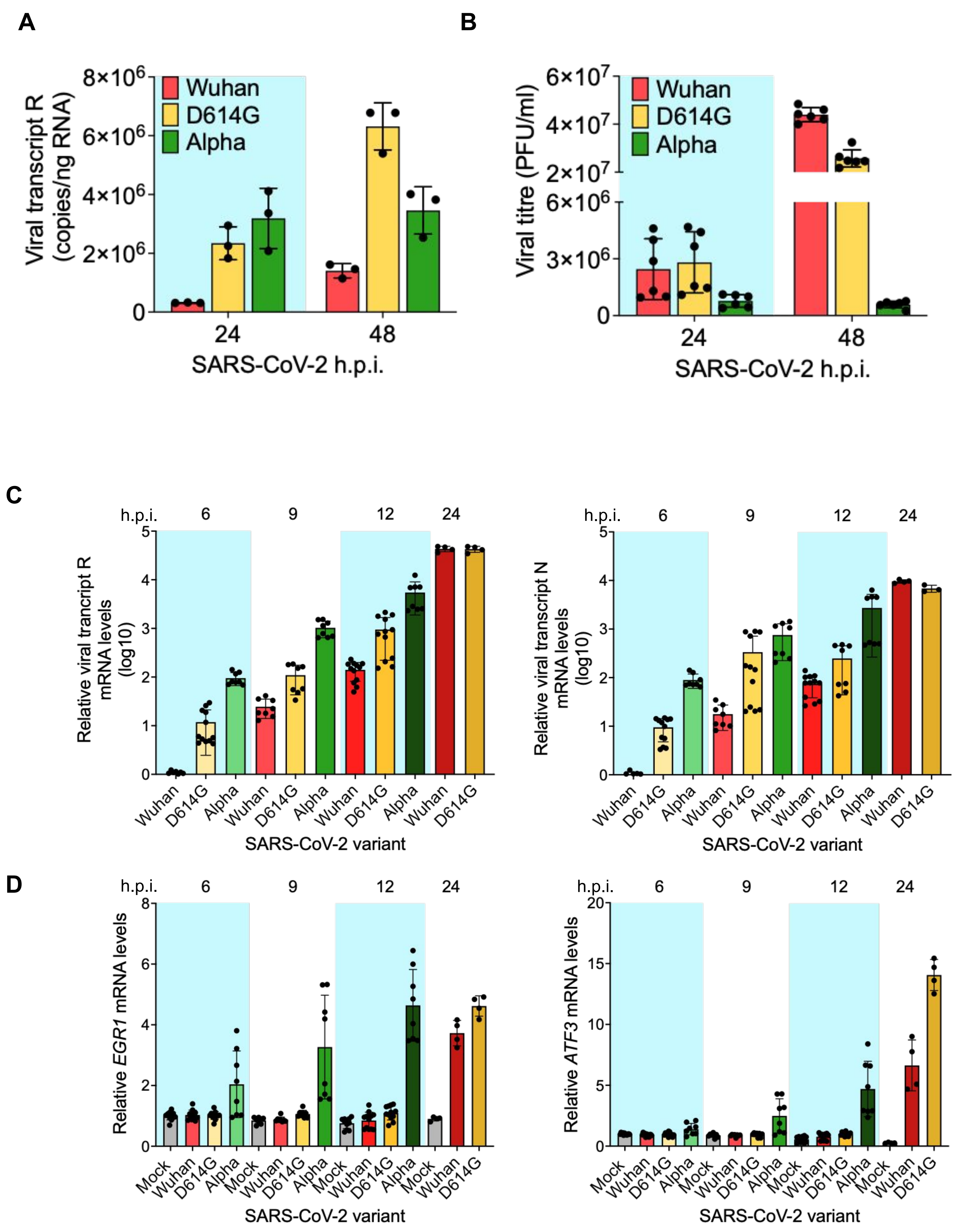

Supplementary Figure S1. SARS-CoV-2 variants' replication kinetics and transcriptional response. Calu-3 cells were infected with the SARS-CoV-2 Wuhan, D614G, and Alpha variants (MOI 0.1). The viral intracellular RNA (**A**) and the viral particles released in the supernatant (**B**) were measured over time (24 and 48 h.p.i.). The RNA copies of the viral transcript R (RNA-dependent RNA polymerase), were calculated using a standard curve generated with a Rpdp-amplicon encoding plasmid. The viral titre was calculated by plaque reduction assay and expressed as PFU/ml. Data are mean ± s.d. of n=3 biological replicates in **A**, and of n=2 biological replicates, each tested in technical triplicate in **B**. Relative expression of (**C**) viral genes (R and N) and (**D**) cellular genes (*EGR1* and *ATF3*) in Calu-3 cells infected with the SARS-CoV-2 Wuhan, D614G, and Alpha variants (MOI 1). Intracellular RNA levels were measured at 6, 9, 12, 24 h.p.i. by qPCR. Data are mean-normalised pooled values from independent experiments (n=2 for Alpha; n=3 for Wuhan and D614G variants). Each condition was tested in 4 replicates per condition in each experiment. EGR1 and ATF3 are genes previously identified by Wyler and colleagues (Wyler et al., 2021) as strongly induced at 12 h.p.i..

SUPPLEMENTARY FIGURE 2

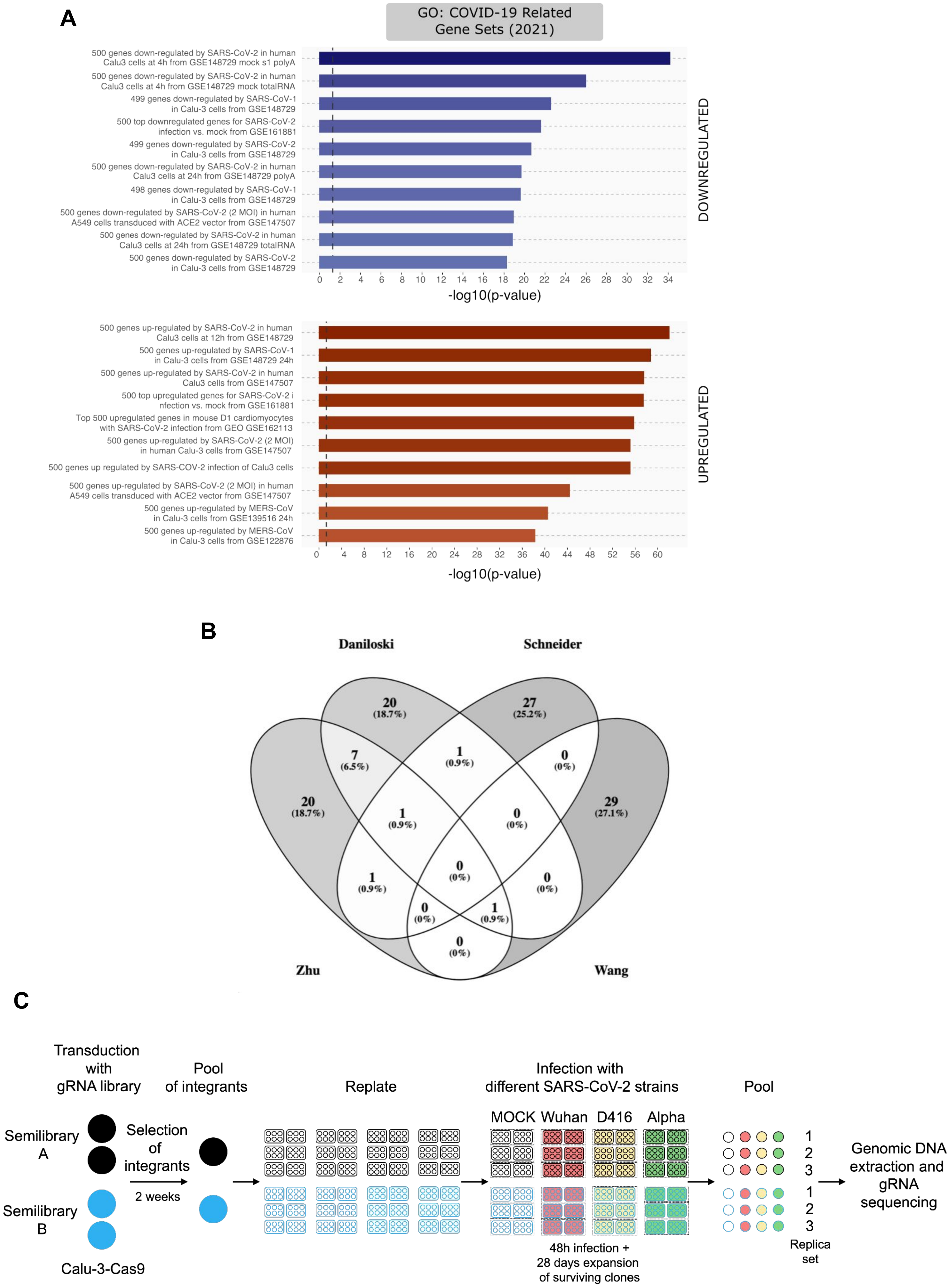

Supplementary Figure S2. CRISPR screening.
(**A**) Gene enrichment analysis on DEGs identified in samples infected with Alpha variant for 12 h.
(**B**) Overlap of cellular genes identified in the different CRISPR-based genetic screens.
(**C**) Workflow of CRISPR-based loss-of-function screening.

SUPPLEMENTARY FIGURE 3

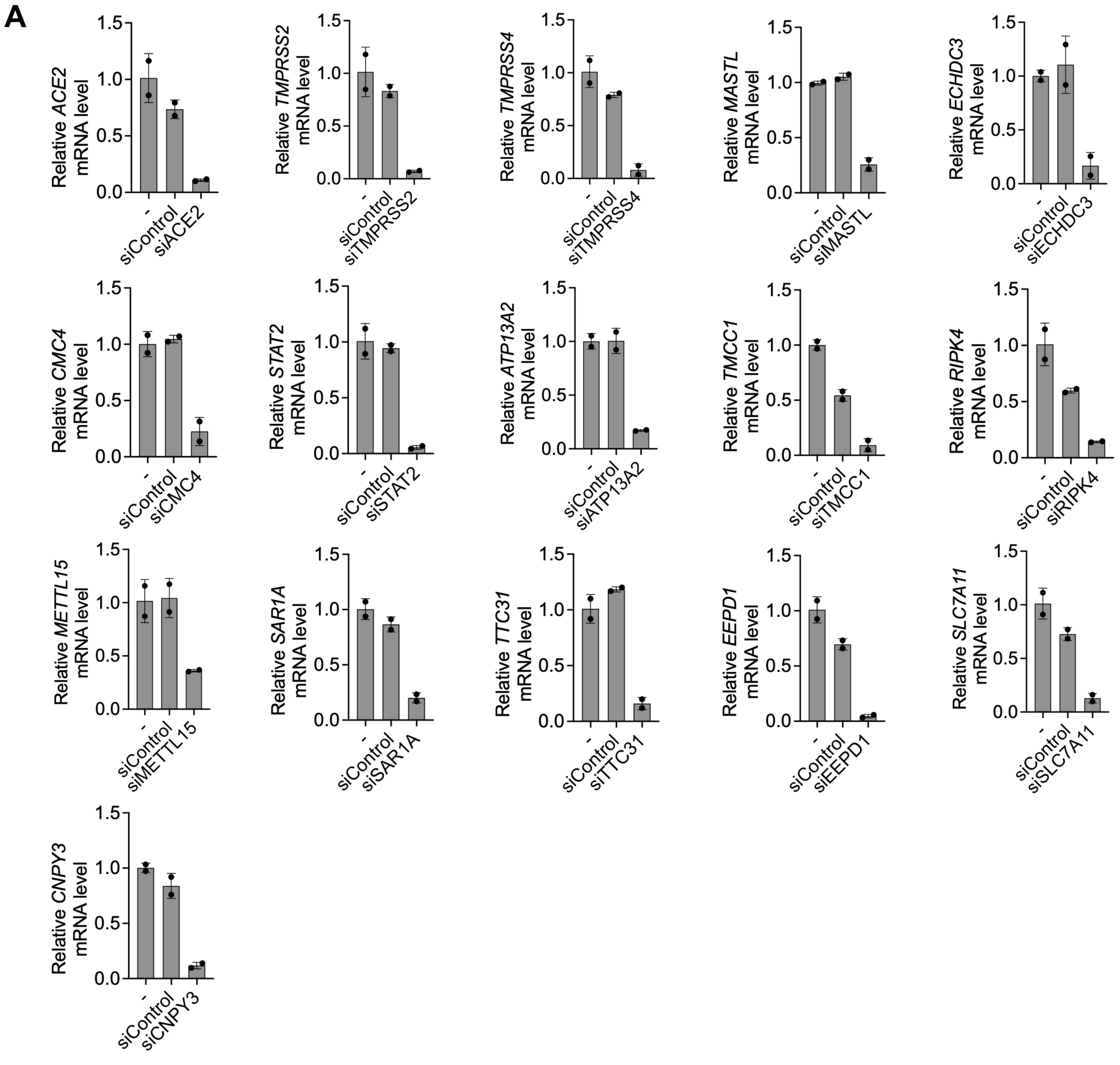

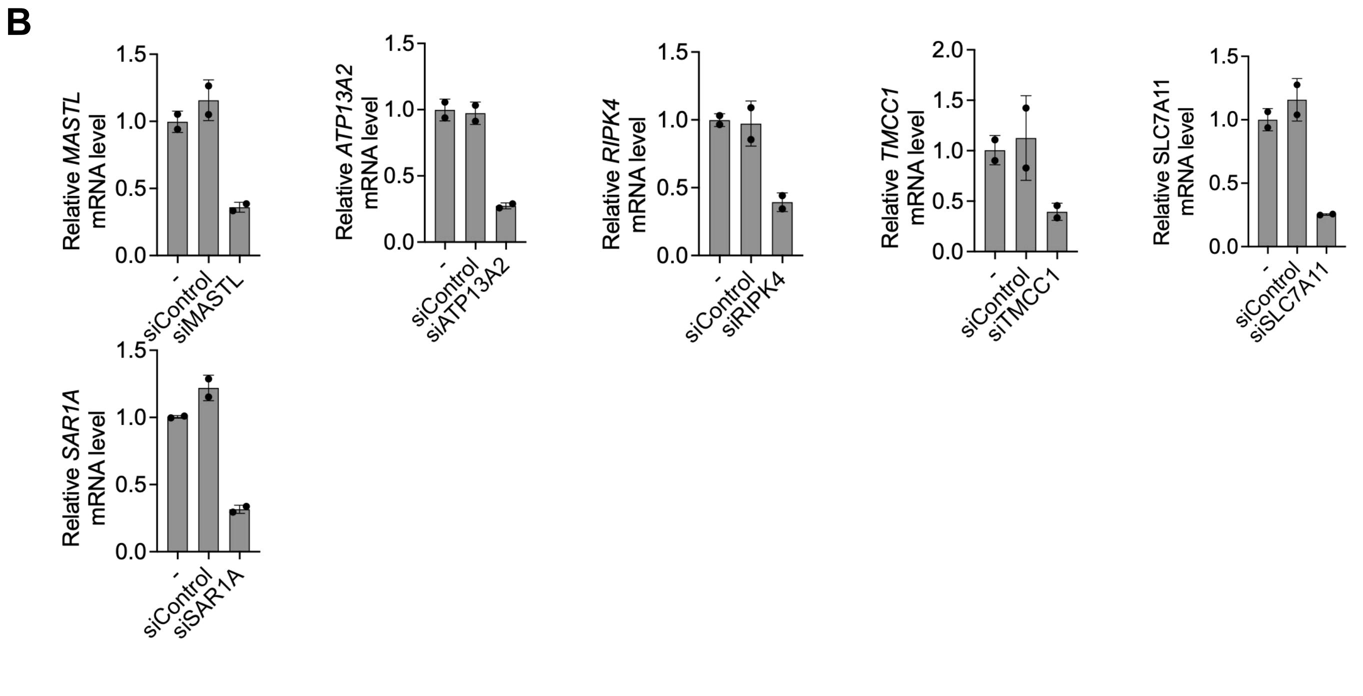

Supplementary Figure S3. siRNA-mediated knock-down efficiency in human cells.
Relative expression of selected candidate hits after siRNA knockdown in (**A**) Calu-3 and (**B**) Caco-2 cell lines. RNA levels were measured by qPCR 48 h post siRNA transfection. Data are mean ± s.d. of n=2 biological replicates.

SUPPLEMENTARY FIGURE 4

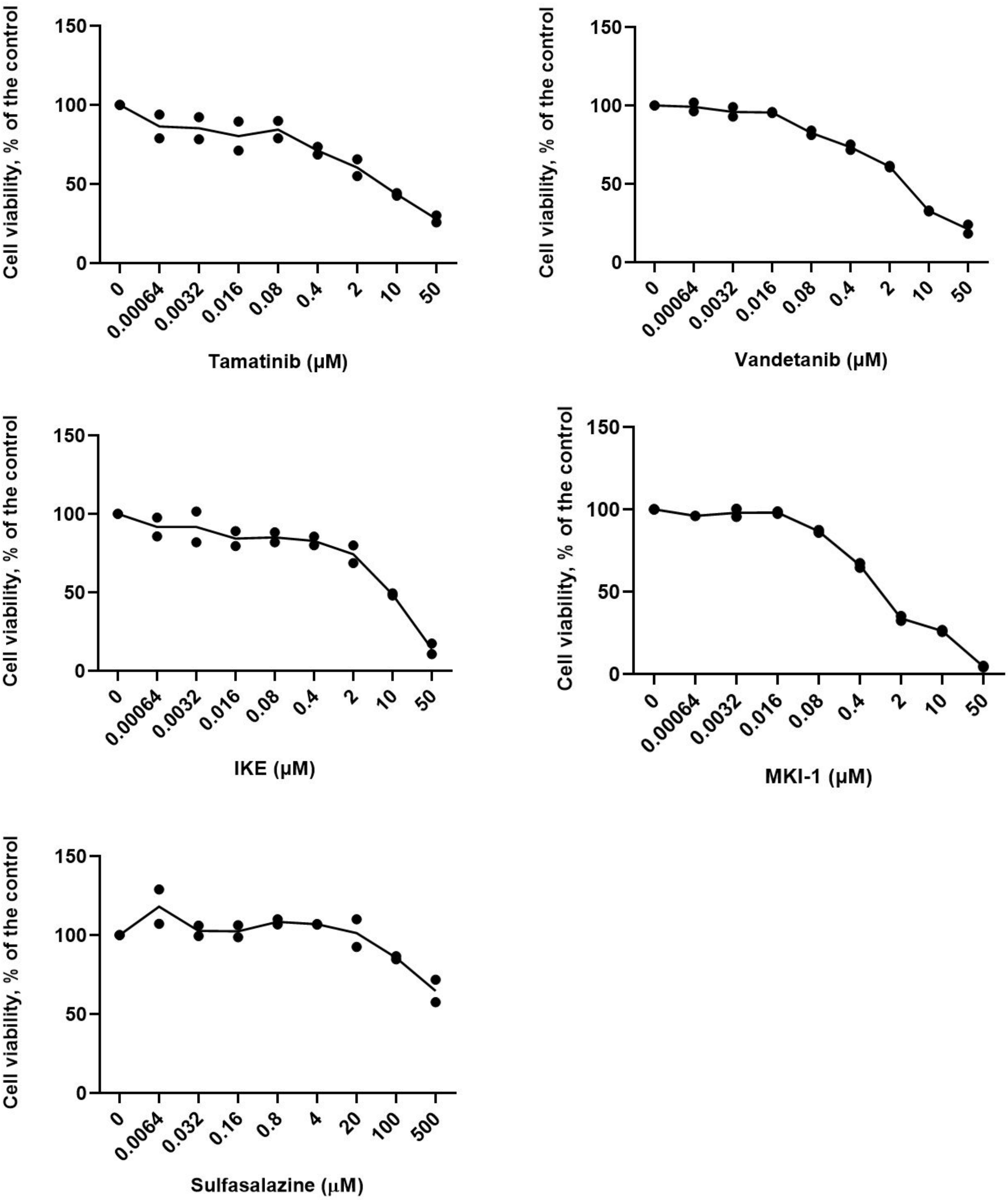

Supplementary Figure S4. Cytotoxicity profiles of tested compounds on Calu-3 cells

Calu-3 cells were seeded and subsequently treated with the tested compounds, at the indicated concentrations, for 48 h. Cell viability was determined by intracellular ATP detection. Mean values of independent experiments (n=2), each conducted in triplicate, are shown as dots. The black line represents the mean dose-response curve.

SUPPLEMENTARY FIGURE 5

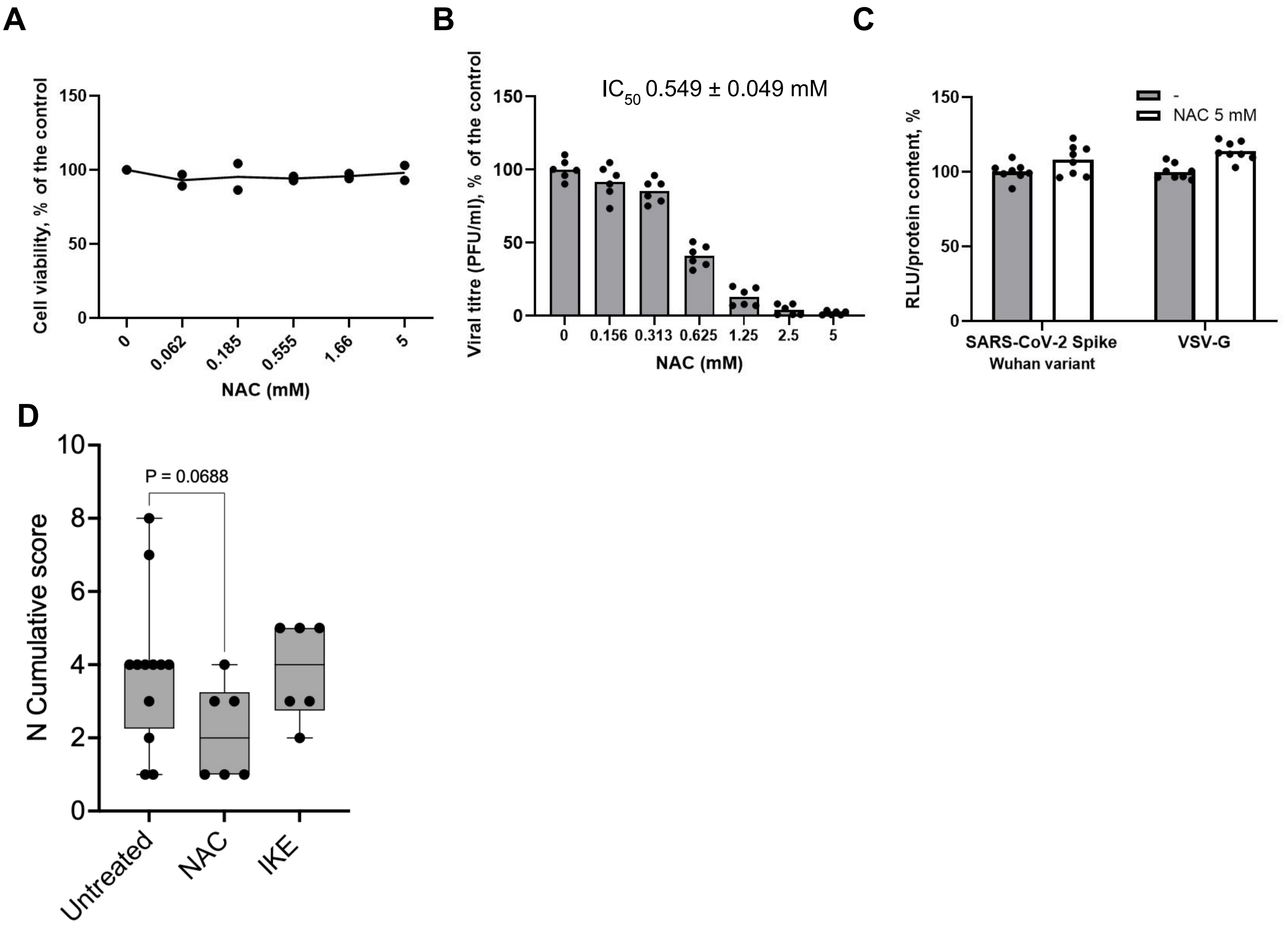

Supplementary Figure S5. Cytotoxicity profile and antiviral properties of NAC in SARS-CoV-2 infected Calu-3 cells

(**A**) Calu-3 cells were seeded and subsequently treated with NAC, at the indicated concentrations, for 48 h. Cell viability was determined by intracellular ATP detection. Mean values of independent experiments (n=2), with four technical replicates, are indicated by dots. The black line represents the mean dose-response curve.

(**B**) Calu-3 cells were treated with NAC h at the indicated concentrations for 24 and infected with SARS-CoV-2 Wuhan variant (MOI 0.1). The compounds were added to the fresh medium 1 h.p.i. and remained in the growth medium throughout the experiment. Two days post infection, the cell medium was subjected to plaque reduction assay and the viral titre was calculated and expressed as PFU/ml. Bars indicate the mean of n=2 biological replicates, each conducted in triplicate. Individual technical replicates are shown as dots.

(**C**) Calu-3 cells were treated with NAC for 24 h with NAC (5 mM) and infected with the SARS-CoV-2 Spike (Wuhan variant) or VSV-G control pseudovirus. Luciferase signals were measured at 24 h.p.i. and normalised to the total protein content. Vehicle-treated cells were used as controls. Bars indicate the mean of n=2 biological replicates, each conducted in quadruplicate. Individual technical replicates are shown as dots.

(**D**) Immunohistochemical analysis of SARS-CoV-2 nucleocapsid protein in a humanised model of SARS-CoV-2 infection treated with NAC and IKE. N cumulative score has been calculated as described in the Methods section and representative images are shown in Figure 6C. Data are presented as whisker plots: midline, median; box, 25–75th percentile; whisker, minimum to maximum values. Test: Kruskal-Wallis with uncorrected Dunn's for multiple comparisons.

References

Chen, E.Y. , Tan, C.M., Kou, Y., et al. (2013). Enrichr: interactive and collabora- tive HTML5 gene list enrichment analysis tool. BMC Bioinf. 14 , 128.

Love, M.I. , Huber, W., and Anders, S. (2014). Moderated estimation of fold change and dispersion for RNA-seq data with DESeq2. Genome Biol. 15 , 550.

Rentsch, M.B. , and Zimmer, G. (2011). A vesicular stomatitis virus replicon- based bioassay for the rapid and sensitive determination of multi-species type I interferon. PLoS One 6 , e25858.

Zangrossi, M. , Chakravarty, P., Romani, P., et al. (2021a). A lung organotypic coculture reveals a role for TFEB-lysosomal axis in the survival of dissemi- nated dormant cancer cells. Cancers *13* , 1007.

Zangrossi, M. , Romani, P., Chakravarty, P., et al. (2021b). EphB6 regulates TFEB-lysosomal pathway and survival of disseminated indolent breast cancer cells. Cancers *13* , 1079.
